# Multipathway Biomarker Profiling of Immunothrombosis in Moderate and Severe COVID-19

**DOI:** 10.3390/jcm15218444

**Published:** 2026-10-29

**Authors:** Giovanni Bousquet, Romaric Lacroix, Laurie Pahus, Sabine Valera, Nathalie Hezard, Laurent Arnaud, Evelyne Abdili, Karine Baumstarck, Damien Barrau, Pascal Chanez, Françoise Dignat-George, Laurent Papazian, Jean-Marie Forel, Sami Hraiech, Pierre Morange, Christophe Guervilly

**Affiliations:** 1 Medical Intensive Care Unit, North Hospital, Assistance Publique-Hôpitaux de Marseille (AP-HM), Chemin des Bourrely, 13015 Marseille, France; sabine.valera@ap-hm.fr (S.V.); damien.barrau@ap-hm.fr (D.B.); laurent.papazian@ap-hm.fr (L.P.); jeanmarie.forel@ap-hm.fr (J.-M.F.); sami.hraiech@ap-hm.fr (S.H.); 2 Centre de Recherche en CardioVasculaire et Nutrition (C2VN), INSERM 1263, Institut National de Recherche pour l’Agriculture, l’Alimentation et l’Environnement (INRAE) 1260, Aix Marseille University, 13005 Marseille, France; romaric.lacroix@ap-hm.fr (R.L.); laurie.pahus@ap-hm.fr (L.P.); pascal.chanez@ap-hm.fr (P.C.); francoise.dignat@ap-hm.fr (F.D.-G.); pierre.morange@ap-hm.fr (P.M.); 3 Hematology Laboratory, La Timone University Hospital of Marseille, 13005 Marseille, France; nathalie.hezard@ap-hm.fr (N.H.); laurent.arnaud@ap-hm.fr (L.A.); evelyne.abdili@ap-hm.fr (E.A.); 4 Clinique des Bronches Allergies et Sommeil, CIC 9502, Hôpital Nord, AP-HM, Aix Marseille University, Chemin des Bourrely, 13015 Marseille, France; 5 CNRS, EFS, ADES, Aix Marseille University, 13015 Marseille, France; 6 Centre d’Etudes et de Recherches sur les Services de Santé et Qualité de vie EA 3279, 13005 Marseille, France; karine.baumstarck@ap-hm.fr; 7 Clinical Research Department, Assistance Publique Hôpitaux de Marseille, 13005 Marseille, France

**Keywords:** COVID-19, immunothrombosis, endothelial dysfunction, extracellular vesicles, biomarkers, coagulation, critical illness

## Abstract

**Background/Objectives**: Coronavirus disease 2019 (COVID-19) is associated with immunothrombosis involving endothelial dysfunction and coagulation activation. We aimed to compare biomarkers spanning multiple immunothrombotic pathways between patients with moderate and severe COVID-19 at baseline and according to clinical outcome. Secondary analyses compared biomarker levels at days 3 and 7. **Methods**: This prospective observational study included 42 hospitalized patients with COVID-19 enrolled between April 2020 and January 2021, comprising 21 with moderate and 21 with severe disease. Biomarkers of coagulation, endothelial dysfunction, endogenous anticoagulant pathways, fibrinolysis, and extracellular vesicle-mediated activity were measured at baseline and on days 3 and 7. The prespecified ventilation/death composite included invasive mechanical ventilation already ongoing at baseline or initiated subsequently and/or death by day 28. **Results**: Seventeen patients (40.5%) met the composite criterion, including 12 patients already receiving invasive mechanical ventilation at baseline. At baseline, severe disease was associated with higher D-dimer (1.74 [0.87–3.67] vs. 0.68 [0.46–0.94] µg/mL FEU), free tissue factor pathway inhibitor (TFPI; 55.00 [38.00–109.00] vs. 16.00 [14.00–29.00] ng/mL), extracellular vesicle-associated tissue factor activity (EV-TF; 28.86 [13.61–48.72] vs. 5.00 [5.00–5.70] fM), and extracellular vesicle-associated plasmin generation capacity (EV-PGC; 93.36 [41.60–303.10] vs. 24.50 [16.14–35.74] fM; all *p* < 0.01). Exploratory ROC analyses further illustrated the separation of these biomarker distributions between the predefined clinical groups. **Conclusions**: Severe COVID-19 and unfavorable outcomes were associated with baseline differences in biomarkers spanning several immunothrombotic pathways, some of which remained detectable at days 3 and 7. Interpretation of the marked TFPI elevation is limited by differences in heparin exposure between severity groups. These exploratory findings require confirmation in larger cohorts, and their incremental value beyond routine clinical assessment remains untested. Outcome analyses largely reflected baseline severity, with only five subsequent events.

## 1. Introduction

Coronavirus disease 2019 (COVID-19) is now recognized as a multisystem thrombo-inflammatory disease in which dysregulated interactions between inflammation, coagulation and the vascular endothelium contribute substantially to disease severity. Beyond viral pneumonia, severe COVID-19 is characterized by a high incidence of both microvascular and macrovascular thrombotic complications despite standard thromboprophylaxis [1,2]. Autopsy studies have demonstrated diffuse pulmonary endothelialitis, extensive microvascular thrombosis and intussusceptive angiogenesis, supporting the central role of vascular injury in organ dysfunction [3]. These manifestations are encompassed by the concept of immunothrombosis, which describes the coordinated interaction between innate immune activation, endothelial dysfunction, platelet activation and coagulation pathway activation [4]. Rather than acting as independent biological processes, these mechanisms constitute an interconnected network that collectively amplifies inflammation, endothelial dysfunction, and thrombosis [4,5,6]. Among these pathways, endothelial dysfunction has emerged as a central driver of severe disease. Several studies have reported disruption of the VWF–ADAMTS13 axis in severe COVID-19, while meta-analytic data support the prognostic relevance of endothelial biomarkers including VWF, angiopoietin-2 and ADAMTS13 activity [7,8,9]. In parallel, conventional coagulation biomarkers, including D-dimer and fibrinogen, have also been associated with disease progression and adverse outcomes [10,11].

Extracellular vesicles (EVs) have emerged as promising integrative biomarkers and potential mediators because they constitute a biological interface between inflammation, endothelial activation and coagulation. By exposing procoagulant phospholipid surfaces and transporting tissue factor (TF) derived from activated vascular and immune cells, EVs actively participate in thrombo-inflammatory processes. Increased extracellular vesicle-associated tissue factor activity (EV-TF) has been associated with disease severity, thrombotic complications and mortality in COVID-19 patients [12,13]. Recent studies have linked EV profiles to COVID-19 severity and prognosis, including TF-positive EVs, inflammatory/thrombogenic EV signatures and platelet–leukocyte EV interactions [14,15,16].

However, most studies have investigated individual components of COVID-19-associated immunothrombosis, thereby providing a fragmented view of a highly interconnected pathophysiological process. Prospective studies simultaneously assessing biomarkers of coagulation activation, endothelial dysfunction, endogenous anticoagulant pathways, fibrinolytic regulation, and extracellular vesicle-mediated activity remain scarce. We therefore aimed primarily to compare a multipathway panel of immunothrombosis biomarkers between patients with moderate and severe COVID-19 at baseline. Secondary objectives were to assess their associations with clinical outcome and to compare biomarker levels at days 3 and 7. Cumulative exposure and longitudinal analyses were performed as additional exploratory analyses.

## 2. Materials and Methods

### 2.1. Study Design and Patients

This prospective observational cohort study was approved by the local ethics committee of Aix-Marseille University (20 April 2020; Ref. 3804-NI). In accordance with French regulations governing non-interventional research, all patients or their legal representatives received complete study information and provided written non-opposition to participation.

Between April 2020 and January 2021, adult patients with confirmed COVID-19 were prospectively enrolled. In accordance with the study design defined a priori, inclusion was continued until 21 patients had been enrolled in each initial severity group (moderate and severe COVID-19).

COVID-19 was diagnosed based on clinical findings consistent with viral pneumonia associated with a positive severe acute respiratory syndrome coronavirus 2 (SARS-CoV-2) reverse transcription polymerase chain reaction (RT-PCR) performed on a nasopharyngeal swab, sputum sample, or bronchoalveolar lavage specimen.

At inclusion, patients were stratified according to their initial respiratory severity. Moderate COVID-19 was defined as a supplemental oxygen requirement of ≤6 L/min. Severe COVID-19 was defined as an oxygen requirement > 6 L/min, the use of high-flow nasal oxygen therapy, or invasive mechanical ventilation at inclusion.

Patients younger than 18 years, pregnant women, individuals deprived of liberty or under legal protection, and patients for whom a decision to withhold or withdraw life-sustaining therapies had already been made were excluded.

### 2.2. Outcomes and Data Collection

The primary objective of the study was to compare baseline biomarker profiles between patients with moderate and severe COVID-19.

Secondary objectives were to compare baseline biomarker profiles according to clinical outcome and to compare biomarker levels between groups at days 3 and 7. Cumulative exposure and longitudinal analyses using models with patient-specific fixed intercepts were performed as additional exploratory analyses.

The prespecified composite criterion was defined as invasive mechanical ventilation, whether already ongoing at baseline or initiated subsequently, and/or death by day 28. Patients already receiving invasive mechanical ventilation at inclusion were therefore classified as meeting the composite criterion at baseline. Throughout the manuscript, the terms “favorable outcome” and “unfavorable outcome” are retained as labels for patients who did not meet and who met this composite criterion, respectively, rather than indicating exclusively subsequent clinical events.

Thromboembolic events present at admission were identified through clinically indicated diagnostic investigations, including computed tomography pulmonary angiography and vascular ultrasonography. No systematic screening protocol for thrombosis was implemented.

Demographic characteristics, body mass index, comorbidities, chronic antiplatelet or anticoagulant therapies, respiratory rate, peripheral oxygen saturation, SARS-CoV-2 RT-PCR cycle threshold values, and chest computed tomography findings were prospectively recorded.

Blood samples were collected within 48 h after hospital admission (day 1) and repeated on days 3 and 7 when patients remained hospitalized.

### 2.3. Biomarker Measurements

Biomarkers were selected to explore complementary biological pathways involved in COVID-19-associated immunothrombosis. They were grouped into five predefined biological domains: coagulation activation, endothelial dysfunction, endogenous anticoagulant pathways, fibrinolytic regulation, and extracellular vesicle-mediated thrombo-inflammatory activity.

Coagulation activation was assessed using D-dimer and fibrinogen levels.

Biomarkers reflecting endothelial dysfunction included von Willebrand factor antigen (VWF:Ag), von Willebrand factor activity (VWF:Act), soluble thrombomodulin (sTM), circulating endothelial cells (CEC), factor VIII, and the VWF:Ag/ADAMTS13 activity ratio, which was used to characterize imbalance of the VWF–ADAMTS13 axis. This ratio was calculated after expressing both measurements in IU/dL, using the formula: [100 × VWF:Ag (IU/mL)]/ADAMTS13 activity (IU/dL).

Biomarkers reflecting endogenous anticoagulant pathways included ADAMTS13, antithrombin, and protein C activities, free protein S antigen, and free tissue factor pathway inhibitor (TFPI) antigen.

Fibrinolytic regulation was assessed using plasminogen activator inhibitor-1 (PAI-1).

EV-TF and extracellular vesicle-associated plasmin generation capacity (EV-PGC) were measured on platelet-free citrated plasma. EV-TF was assessed using the optimized assay previously described by Vallier et al. and subsequently applied in COVID-19 patients by Guervilly et al. [12,17].

Detailed analytical procedures are provided in Appendix A.

### 2.4. Anticoagulation Management

Thromboprophylaxis was standardized according to the recommendations of the French Society on Thrombosis and Haemostasis available at the time of the study [18].

Patients with moderate COVID-19 received low-molecular-weight heparin (LMWH) at a dose of 4000 IU once daily. Patients with severe COVID-19 and/or obesity received LMWH at a dose of 4000 IU twice daily.

Patients diagnosed with thromboembolic events or requiring extracorporeal membrane oxygenation received therapeutic unfractionated heparin targeting an anti-factor Xa activity between 0.3 and 0.6 IU/mL.

When thromboembolic complications were clinically suspected and/or markedly elevated D-dimer levels were observed, additional investigations, including computed tomography pulmonary angiography or vascular ultrasonography, were performed.

Actual anticoagulant treatment was recorded at each study visit (days 1, 3, and 7), including the anticoagulant agent, prescribed dose, and treatment intensity (standard prophylactic, intensified prophylactic, or therapeutic anticoagulation). Chronic anticoagulant use, extracorporeal membrane oxygenation, and anti-factor Xa activity were also documented.

### 2.5. Statistical Analysis

This exploratory prospective study used a predefined convenience sample of patients enrolled during the study period, with inclusion continuing until 21 patients had been included in each initial severity group. No formal sample size calculation was performed because preliminary data were unavailable for several biomarkers investigated.

Two predefined comparisons were performed throughout the study: (1) moderate versus severe COVID-19 at inclusion and (2) favorable versus unfavorable outcomes.

Continuous variables were expressed as mean ± standard deviation or median (interquartile range), as appropriate. Categorical variables were expressed as number (percentage). Values reported as below or above an assay reporting limit were entered at the corresponding limit for analysis. Between-group comparisons were performed using the Mann–Whitney U test for continuous variables. For categorical variables, Pearson’s χ^2^ test without continuity correction was used when all expected cell counts were at least 5; otherwise, an exact test was used (two-sided Fisher’s exact test for 2 × 2 tables and its extension for larger contingency tables). Variables with no variability were described without hypothesis testing.

Primary cross-sectional analyses compared baseline biomarker values between patients with moderate and severe COVID-19 using the Mann–Whitney U test. Baseline biomarker values were also compared according to favorable or unfavorable clinical outcome. Receiver operating characteristic (ROC) analyses were performed as exploratory descriptive measures of the separation between biomarker distributions across the predefined clinical groups, rather than to evaluate diagnostic or prognostic performance. Initial disease severity was already established according to respiratory support requirements, and the unfavorable-outcome group included patients receiving invasive mechanical ventilation at baseline. Therefore, these analyses were not intended to assess incremental clinical value or predict subsequent deterioration. Areas under the ROC curves (AUCs) and their 95% confidence intervals (95% CIs) were estimated using DeLong’s method. For all ROC analyses, severe disease and unfavorable outcome were defined as the positive classes, and higher biomarker values were consistently assigned to the positive direction, without data-dependent reversal. Accordingly, AUC values below 0.5 indicate that lower biomarker values were associated with the positive class.

A post hoc exploratory analysis was restricted to patients who had not already reached the composite endpoint at the first blood sample, excluding those already receiving invasive mechanical ventilation. Baseline biomarker values were compared descriptively between patients with and without a subsequently documented composite event. Medians, interquartile ranges and available sample sizes were reported, without additional hypothesis tests or ROC analyses because of the small number of events.

An additional exploratory sensitivity analysis compared baseline free TFPI concentrations between severity groups among patients receiving the same intensified prophylactic regimen of enoxaparin (4000 IU twice daily), using an exact two-sided Mann–Whitney U test.

Secondary cross-sectional comparisons were performed separately at days 3 and 7 using the Mann–Whitney U test. Exploratory ROC analyses at days 3 and 7 are reported in Tables S5 and S6.

As an additional exploratory analysis, time-weighted mean biomarker levels were calculated over each patient’s available observation period within the first week of hospitalization. The trapezoidal area under the concentration–time curve was divided by the interval between the first and last available measurements. Only patients with at least two available measurements for the corresponding biomarker were included. These measures summarize biomarker levels over the observed interval, which could differ between patients, and do not necessarily represent exposure over the entire first week. Between-group comparisons were performed using the Mann–Whitney U test.

In a separate exploratory analysis, biomarker values obtained at days 1, 3 and 7 were analyzed using linear models fitted by ordinary least squares, with a patient-specific fixed intercept. Time was treated as a categorical variable, with day 1 as the reference time point. Models included time effects and time-by-group interaction terms, while time-invariant differences between patients were captured by the patient-specific intercepts. The overall time-by-group interaction was assessed using an F test comparing nested models with and without the interaction terms to determine whether changes in biomarker levels differed between groups. Models were fitted using all available measurements, without imputation of missing values. These analyses were considered exploratory.

Missing data resulting from hospital discharge or death were not imputed. All analyses were considered exploratory, and no adjustment for multiple testing was applied. Reported *p* values are therefore nominal and should be interpreted in the context of the large number of comparisons. A two-sided *p* value < 0.05 was used to flag exploratory associations rather than to provide confirmatory evidence.

Statistical analyses were performed using R (version 4.6.1; R Foundation for Statistical Computing, Vienna, Austria) in the RStudio environment (version 2026.08.2; Posit Software, Boston, MA, USA).

Generative artificial intelligence (ChatGPT, GPT-5.6 Sol; OpenAI, San Francisco, CA, USA) was used to verify selected statistical calculations and to assist in checking the consistency of the statistical analyses, results, and their interpretation. All statistical analyses were performed by the authors, who critically reviewed and validated all AI-assisted outputs.

## 3. Results

### 3.1. Patient Characteristics

Forty-two patients were prospectively included in the study. In accordance with the predefined study design, the study population comprised 21 patients (50%) with moderate COVID-19 and 21 patients (50%) with severe COVID-19 according to respiratory support requirements at inclusion. Seventeen patients (40.5%) were classified as having an unfavorable outcome, including 12 patients who were already receiving invasive mechanical ventilation at inclusion. Twenty-five patients (59.5%) had a favorable clinical course.

Baseline demographic, clinical and laboratory characteristics are summarized in Table 1. No statistically significant between-group differences were observed in age, sex distribution, body mass index or the comorbidities assessed. Chronic anticoagulation therapy was more frequent in patients with severe COVID-19 than in those with moderate disease (5/21, 23.8% vs. 0/21, 0%; *p* = 0.048). Actual anticoagulant regimens at baseline differed substantially between severity groups. Among patients with moderate COVID-19, 8/21 (38.1%) received standard prophylaxis, 7/21 (33.3%) intensified prophylaxis, and 6/21 (28.6%) received therapeutic anticoagulation. Among patients with severe COVID-19, the corresponding numbers were 1/21 (4.8%), 3/21 (14.3%), and 15/21 (71.4%), respectively, while no anticoagulant regimen was documented at the day 1 visit for two patients. Continuous intravenous unfractionated heparin was recorded in 13/21 (61.9%) patients with severe disease and none of those with moderate disease. Two patients with severe COVID-19 were receiving venovenous extracorporeal membrane oxygenation at baseline. Anticoagulant agents and treatment intensities documented at days 1, 3, and 7 are summarized in Supplementary Table S9.

At admission, patients with severe COVID-19 required higher levels of respiratory support, had lower PaO_2_/FiO_2_ ratios than patients with moderate disease (180 [131–240] vs. 329 [277–403] mmHg, *p* < 0.001) and higher SOFA scores (4 [3–7.25] vs. 1 [1–2], *p* < 0.001). They also had higher PaCO_2_ values (37.00 [35.00–45.00] vs. 34.00 [32.50–36.50] mmHg, *p* = 0.02) and more extensive radiological involvement, with ground-glass opacities involving more than 50% of lung parenchyma more frequently observed in severe patients among those with available chest CT (9/12, 75.0% vs. 2/21, 9.5%; *p* < 0.001).

Biological variables at inclusion showed higher leukocyte counts in severe patients (8.30 [7.00–15.00] vs. 6.10 [5.30–7.10] G/L, *p* = 0.01), lower prothrombin activity (85.00 [70.00–99.00] vs. 96.50 [93.00–106.50]%, *p* < 0.01), higher activated partial thromboplastin time ratios (1.40 [1.20–1.80] vs. 1.10 [1.00–1.20], *p* < 0.001) and higher C-reactive protein levels (179.95 [123.15–270.15] vs. 78.30 [26.55–161.55] mg/L, *p* < 0.01), whereas platelet counts did not differ between groups (270 [172–344] vs. 245.5 [208–294] G/L, *p* = 0.80).

During hospitalization, 25 patients (59.5%) were admitted to the intensive care unit, including 21/21 patients (100%) from the severe group and 4/21 patients (19.0%) from the moderate group. Sixteen patients (38.1%) required invasive mechanical ventilation, all from the severe group (16/21, 76.2%). Twelve patients were already ventilated at the first blood sample: nine had been intubated before the sampling date, at −7 days (*n* = 1), −3 days (*n* = 4) or −1 day (*n* = 4), and three on the sampling date. Four patients were subsequently intubated at +1 day (*n* = 3) or +8 days (*n* = 1). Three patients (7.1%) died by day 28, including 2/21 patients (9.5%) from the severe group and 1/21 patient (4.8%) from the moderate group. Deaths occurred 10, 10 and 13 days after the first blood sample. The patient who died at +13 days was ventilated at baseline. Of the two patients who died at +10 days, one had been intubated at +1 day, and the other had not received invasive ventilation. ICU and hospital lengths of stay were longer in severe patients than in moderate patients, with median ICU length of stay of 15 [9–28] vs. 0 [0–0] days (*p* < 0.001) and median hospital length of stay of 28 [15–32] vs. 8 [6–14] days (*p* < 0.001), respectively.

At admission, computed tomography pulmonary angiography was performed in 33 of 42 patients (78.6%), and vascular ultrasonography was performed in one patient. Five thromboembolic events were documented in four patients (9.5%): three pulmonary emboli, one ischemic stroke, and one acute lower limb arterial ischemia. One patient had both pulmonary embolism and ischemic stroke. Four events were diagnosed on the same calendar day as the first blood sample, while one pulmonary embolism was diagnosed the preceding day.

### 3.2. Biomarker Profiles at Admission According to Initial Disease Severity

Baseline biomarker profiles according to initial disease severity are summarized in Table 2.

At admission, within the coagulation activation compartment, D-dimer concentrations were higher in patients with severe COVID-19 than in those with moderate disease (1.74 [0.87–3.67] vs. 0.68 [0.46–0.94] µg/mL FEU, *p* < 0.01), whereas fibrinogen concentrations did not significantly differ between groups (7.10 [5.99–7.84] vs. 6.58 [5.77–7.31] g/L, *p* = 0.30).

Within the endothelial dysfunction compartment, sTM, VWF:Ag, factor VIII, and the VWF:Ag/ADAMTS13 ratio were higher in patients with severe disease. Median sTM concentrations were 4826.60 [4031.45–7335.93] pg/mL in severe patients compared with 3771.65 [3198.88–4459.42] pg/mL in moderate patients (*p* < 0.01). Similarly, VWF:Ag concentrations were higher in severe patients (4.05 [3.09–4.20] vs. 3.18 [2.71–3.50] IU/mL, *p* < 0.01), as were factor VIII levels (2.69 [1.97–3.05] vs. 2.04 [1.62–2.29] IU/mL, *p* = 0.03) and the VWF:Ag/ADAMTS13 ratio (5.19 [3.77–7.96] vs. 3.87 [2.87–4.72], *p* < 0.01). No significant differences were observed for circulating endothelial cells (5.00 [1.00–16.00] vs. 3.00 [0.50–15.50] cells/mL, *p* = 0.71) or VWF:Act (3.20 [2.31–3.83] vs. 2.70 [2.48–3.00] IU/mL, *p* = 0.32).

Within the endogenous anticoagulant pathway compartment, ADAMTS13 and protein C activities were lower in patients with severe disease. Median ADAMTS13 activity was 69.00 [56.00–84.00] IU/dL in severe patients compared with 85.00 [78.50–98.00] IU/dL in moderate patients (*p* < 0.01), while protein C activity was 80.00 [75.00–107.00] versus 96.00 [84.50–115.00]%, respectively (*p* = 0.02). Antithrombin activity was numerically lower in severe patients, although the difference did not reach statistical significance (96.00 [80.00–106.00] vs. 102.00 [94.50–114.00]%, *p* = 0.06). Free protein S antigen levels did not differ significantly between groups (79.00 [65.00–101.00] vs. 79.00 [71.50–100.00]%, *p* = 0.66). In contrast, free TFPI antigen concentrations were markedly higher in patients with severe disease (55.00 [38.00–109.00] vs. 16.00 [14.00–29.00] ng/mL, *p* < 0.001).

Within the fibrinolytic regulation compartment, PAI-1 concentrations did not significantly differ between severe and moderate patients (89.00 [61.00–141.00] vs. 87.00 [73.00–100.50] ng/mL, *p* = 0.80).

Within the extracellular vesicle-mediated thrombo-inflammatory activity compartment, both EV-TF and EV-PGC were higher in patients with severe disease. Median EV-TF activity increased from 5.00 [5.00–5.70] fM in moderate patients to 28.86 [13.61–48.72] fM in severe patients (*p* < 0.001), while EV-PGC increased from 24.50 [16.14–35.74] to 93.36 [41.60–303.10] fM (*p* < 0.001).

**Figure 1:**
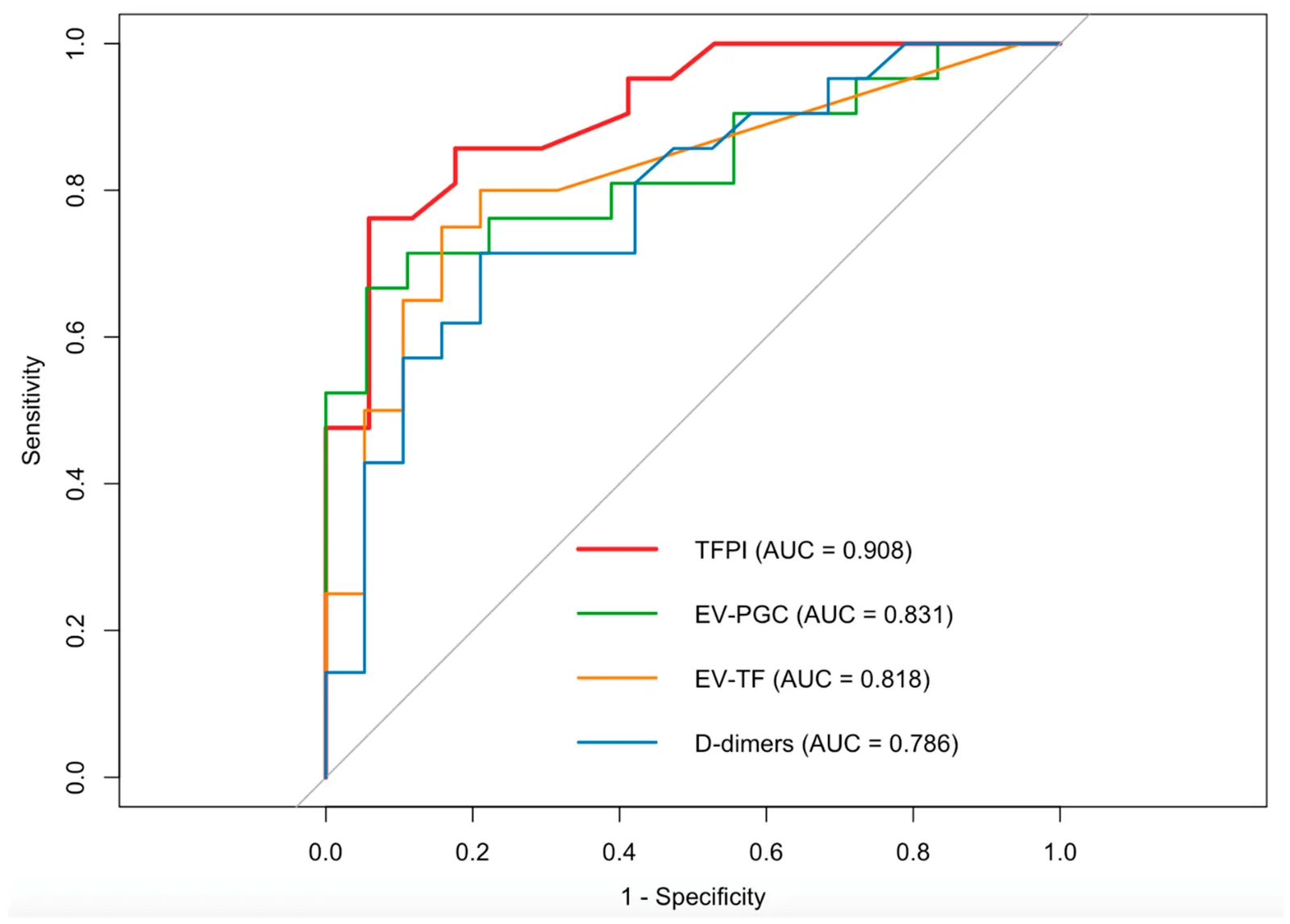
Receiver operating characteristic (ROC) curves of biomarkers at admission for initial disease severity. Sample sizes varied according to biomarker availability: TFPI, *n* = 38 (17 moderate, 21 severe); EV-PGC, *n* = 39 (18 moderate, 21 severe); EV-TF, *n* = 39 (19 moderate, 20 severe); and D-dimer, *n* = 40 (19 moderate, 21 severe).

Exploratory ROC analyses were used to describe the separation of baseline biomarker distributions between the predefined severity groups. For TFPI, EV-PGC, EV-TF, and D-dimer, the AUCs (95% CIs) were 0.908 (0.816–0.999), 0.831 (0.700–0.961), 0.818 (0.687–0.950), and 0.786 (0.642–0.929), respectively (Figure 1).

In an exploratory sensitivity analysis restricted to patients receiving the same intensified prophylactic regimen of enoxaparin (4000 IU twice daily), baseline free TFPI concentrations remained numerically higher in patients with severe COVID-19 (median 35.0 ng/mL, *n* = 3) than in those with moderate COVID-19 (median 14.5 ng/mL, *n* = 4). However, this difference was not statistically significant (exact two-sided Mann–Whitney U test, *p* = 0.114). This analysis was limited by the very small sample size and unavailable information on treatment duration and timing relative to blood sampling.

### 3.3. Biomarker Profiles at Admission According to Clinical Outcome

To assess whether admission biomarker profiles differed between patients with favorable and unfavorable outcomes, baseline biomarker concentrations were compared between the two groups. These results are summarized in Table 3.

At admission, within the coagulation activation compartment, D-dimer concentrations were higher in patients with unfavorable outcomes than in those with favorable outcomes (1.98 [1.38–3.95] vs. 0.60 [0.46–0.94] µg/mL FEU, *p* < 0.001), whereas fibrinogen concentrations did not significantly differ between groups (7.30 [6.21–8.80] vs. 6.24 [5.51–7.21] g/L, *p* = 0.06).

Within the endothelial dysfunction compartment, sTM, VWF:Ag and the VWF:Ag/ADAMTS13 ratio were higher in patients with unfavorable outcomes. Median sTM concentrations were 5665.35 [4119.95–9167.40] pg/mL in patients with unfavorable outcomes compared with 3927.80 [3312.95–4644.65] pg/mL in those with favorable outcomes (*p* < 0.01). VWF:Ag concentrations were also higher (4.05 [3.32–4.20] vs. 3.09 [2.71–3.66] IU/mL, *p* = 0.01), as was the VWF:Ag/ADAMTS13 ratio (5.65 [4.29–9.05] vs. 3.72 [3.02–4.72], *p* < 0.01). No significant differences were observed for circulating endothelial cells, VWF:Act or factor VIII.

Within the endogenous anticoagulant pathway compartment, ADAMTS13, antithrombin, and protein C activities were lower in patients with unfavorable outcomes. Median ADAMTS13 activity was 66.00 [47.00–88.00] IU/dL in patients with unfavorable outcomes compared with 83.00 [75.50–98.00] IU/dL in those with favorable outcomes (*p* < 0.01). Median antithrombin activity was 82.00 [73.00–99.00]% in patients with unfavorable outcomes compared with 104.00 [96.50–115.50]% in those with favorable outcomes (*p* < 0.01), while protein C activity was 76.00 [66.00–96.00]% versus 106.00 [84.50–115.00]%, respectively (*p* < 0.01). Free protein S antigen levels did not differ significantly between groups (79.00 [65.00–101.00] vs. 79.00 [68.50–100.00]%, *p* = 0.97). Free TFPI antigen concentrations remained markedly higher in patients with unfavorable outcomes (57.00 [40.00–112.00] vs. 25.00 [14.00–32.00] ng/mL, *p* < 0.001).

No significant difference in PAI-1 concentrations was observed between outcome groups (86.00 [61.00–141.00] vs. 89.00 [73.00–109.00] ng/mL, *p* = 0.99).

Within the extracellular vesicle-mediated thrombo-inflammatory activity compartment, both EV-TF and EV-PGC were higher in patients with unfavorable outcomes. Median EV-TF activity was 30.27 [14.23–59.08] fM in patients with unfavorable outcomes compared with 5.00 [5.00–5.96] fM in those with favorable outcomes (*p* < 0.001), while EV-PGC values were 102.16 [66.62–376.05] and 24.57 [16.59–42.06] fM, respectively (*p* < 0.001).

**Figure 2:**
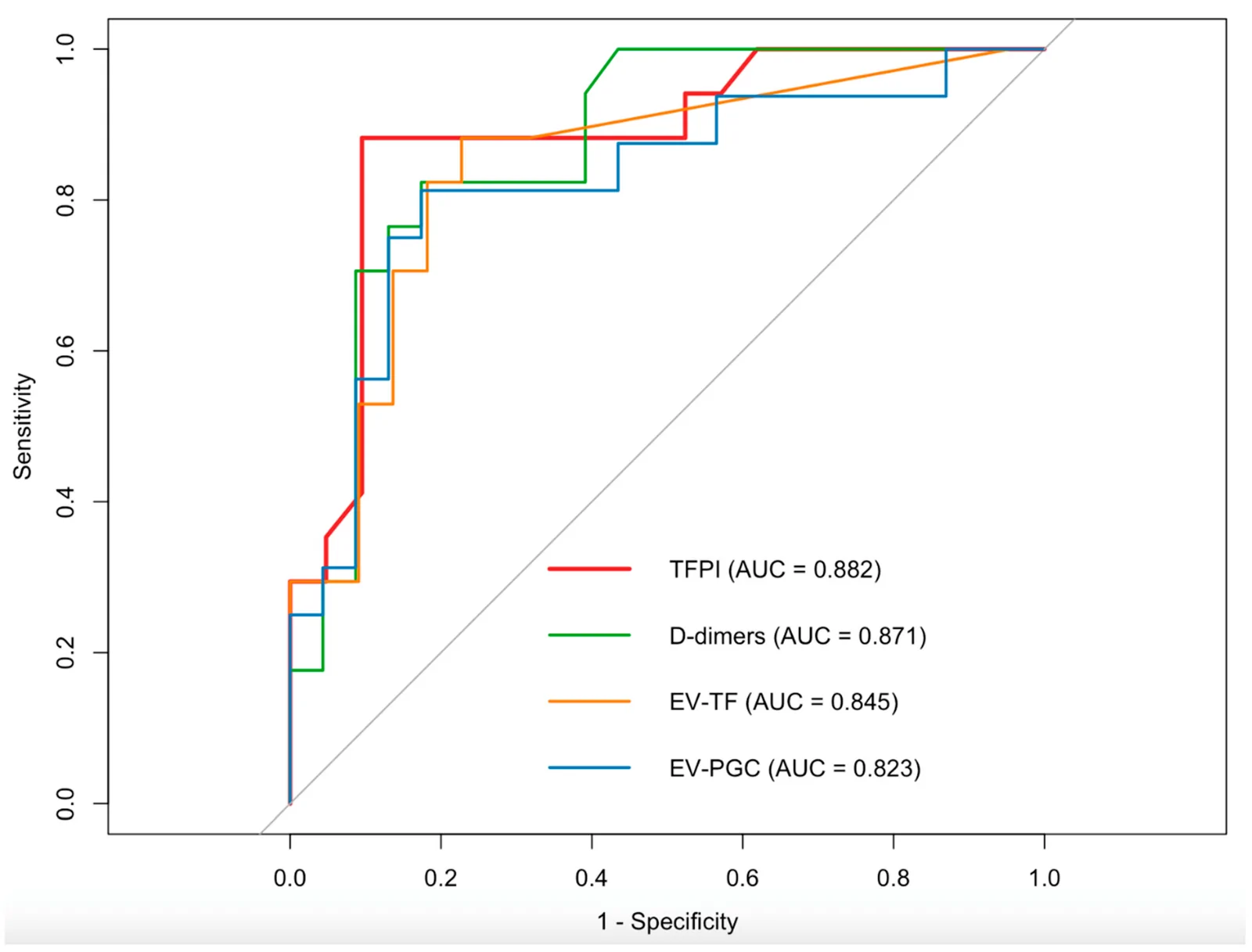
Receiver operating characteristic (ROC) curves of biomarkers at admission for unfavorable outcomes. Sample sizes varied according to biomarker availability: TFPI, *n* = 38 (21 favorable, 17 unfavorable); D-dimer, *n* = 40 (23 favorable, 17 unfavorable); EV-TF, *n* = 39 (22 favorable, 17 unfavorable); and EV-PGC, *n* = 39 (23 favorable, 16 unfavorable).

Exploratory ROC analyses were also performed to describe the separation of baseline biomarker distributions between patients classified according to clinical outcome. For TFPI, D-dimer, EV-TF, and EV-PGC, the AUCs (95% CIs) were 0.882 (0.767–0.997), 0.871 (0.759–0.983), 0.845 (0.719–0.971), and 0.823 (0.680–0.967), respectively (Figure 2).

In the post hoc exploratory analysis excluding the 12 patients already receiving invasive mechanical ventilation at the first blood sample, 30 patients remained. Five subsequently reached the composite endpoint: four required invasive mechanical ventilation, including one who subsequently died, and one died without invasive mechanical ventilation. The remaining 25 patients had no documented composite event. Baseline biomarker distributions are presented in Supplementary Table S8.

### 3.4. Biomarker Profiles at Days 3 and 7

**Figure 3:**
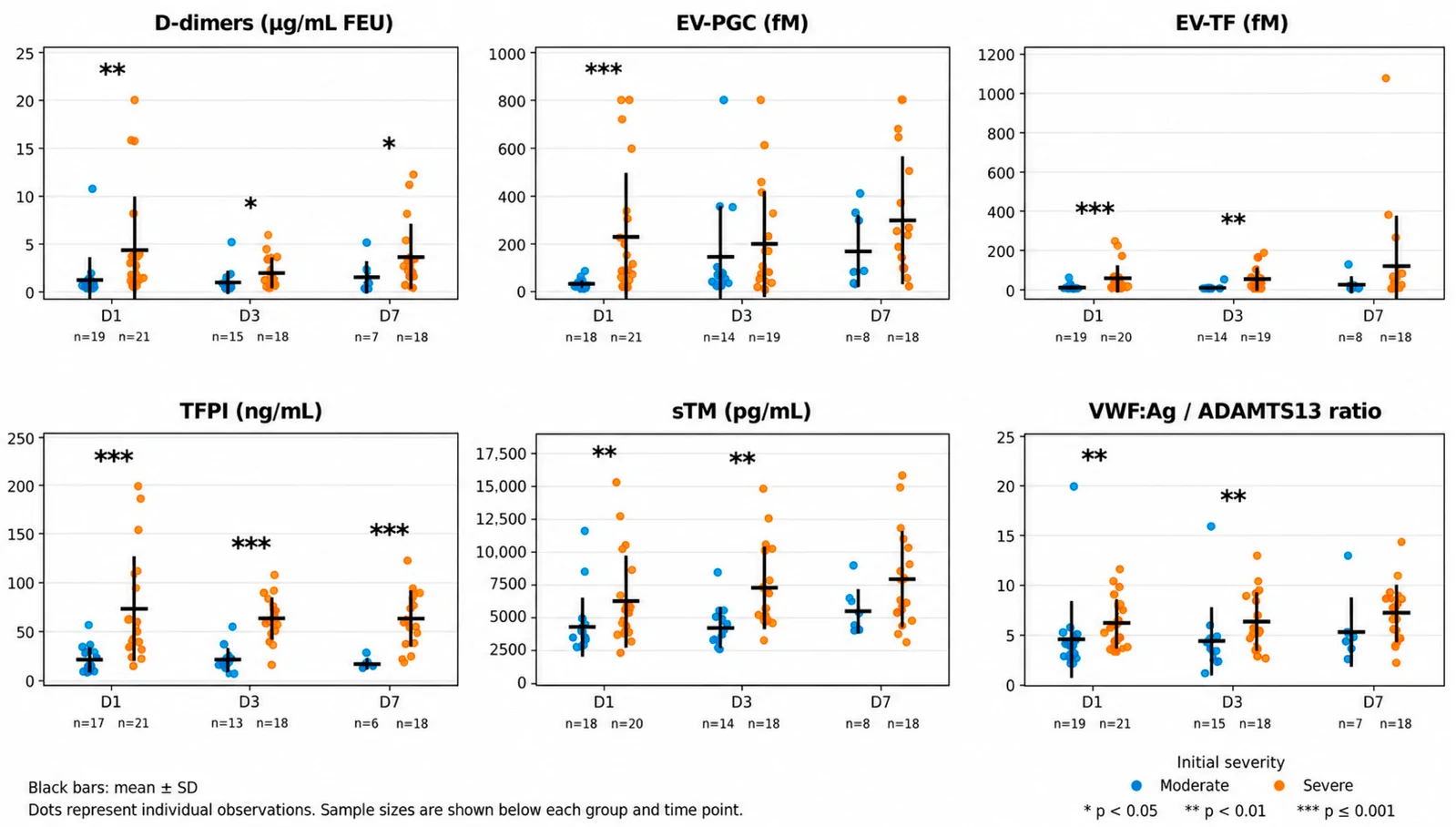
Biomarker values at baseline and on days 3 and 7 according to initial disease severity. Dots represent individual observations, and black bars indicate mean ± standard deviation. Sample sizes are shown below each group and time point. Asterisks indicate pointwise between-group differences assessed using the Mann–Whitney U test (* *p* < 0.05, ** *p* < 0.01, *** *p* ≤ 0.001). All pointwise *p* values are nominal and were not adjusted for multiple comparisons. Sample availability varied across time points, and these cross-sectional comparisons should not be interpreted as within-patient trajectories.

According to initial disease severity, several differences observed at admission persisted at day 3. Compared with patients with moderate disease, patients with severe COVID-19 had higher D-dimer concentrations (1.26 [0.64–3.40] vs. 0.73 [0.44–0.82] µg/mL FEU, *p* = 0.047), sTM concentrations (6443.55 [5136.65–9709.55] vs. 4023.40 [3421.28–5156.65] pg/mL, *p* < 0.01), VWF:Ag levels (4.10 [3.32–4.91] vs. 3.10 [2.66–3.79] IU/mL, *p* = 0.02), and VWF:Ag/ADAMTS13 ratios (5.43 [4.07–8.74] vs. 3.41 [2.83–4.55], *p* < 0.01). ADAMTS13 activity was lower in severe patients (70.00 [60.25–87.00] vs. 87.00 [82.00–98.00] IU/dL, *p* = 0.02), whereas TFPI concentrations (60.00 [51.25–74.75] vs. 21.00 [15.00–25.00] ng/mL, *p* < 0.001) and EV-TF activity (31.03 [7.02–64.06] vs. 5.00 [5.00–5.00] fM, *p* < 0.01) remained markedly higher. At day 7, severe patients continued to have higher D-dimer concentrations (2.55 [1.66–3.50] vs. 0.87 [0.35–1.94] µg/mL FEU, *p* = 0.03), fibrinogen levels (6.79 [5.52–8.54] vs. 5.07 [4.51–5.47] g/L, *p* = 0.046), VWF:Act (4.58 [3.98–5.15] vs. 2.43 [2.10–3.27] IU/mL, *p* < 0.01), VWF:Ag (4.71 [4.20–6.30] vs. 2.84 [2.73–3.16] IU/mL, *p* < 0.01), and TFPI concentrations (61.00 [41.50–88.00] vs. 16.00 [14.25–19.25] ng/mL, *p* < 0.001). EV-TF and EV-PGC values remained numerically higher in severe patients at day 7, but the differences were no longer statistically significant. These results are summarized in Tables S1 and S2, with selected biomarker profiles illustrated in Figure 3.

**Figure 4:**
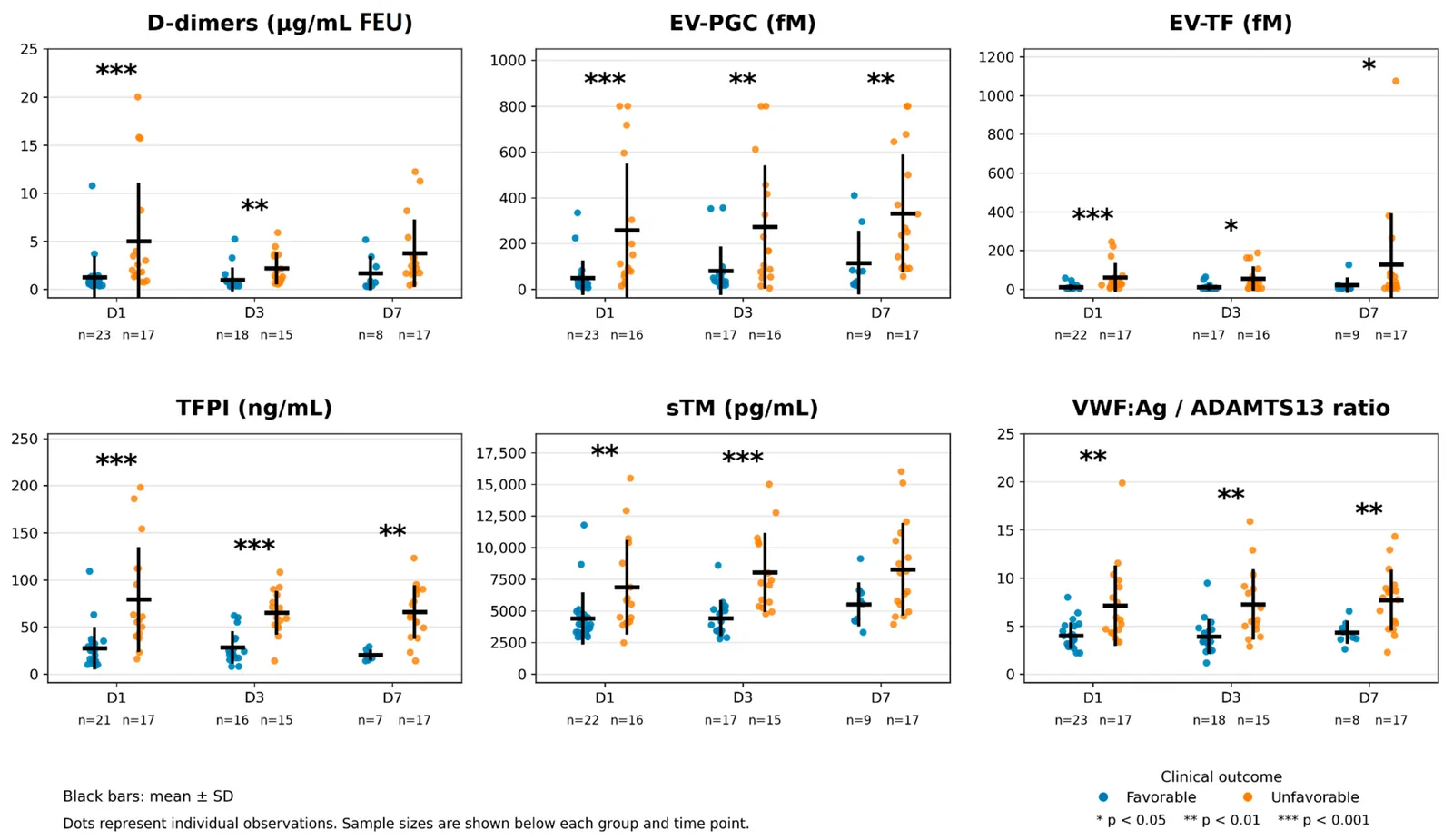
Biomarker values at baseline and on days 3 and 7 according to clinical outcome. Dots represent individual observations, and black bars indicate mean ± standard deviation. Sample sizes are shown below each group and time point. Asterisks indicate pointwise between-group differences assessed using the Mann–Whitney U test (* *p* < 0.05, ** *p* < 0.01, *** *p* ≤ 0.001). All pointwise *p* values are nominal and were not adjusted for multiple comparisons. Sample availability varied across time points, and these cross-sectional comparisons should not be interpreted as within-patient trajectories.

According to clinical outcome, broader between-group differences were observed. At day 3, patients with an unfavorable outcome had higher D-dimer concentrations (1.42 [0.88–3.52] vs. 0.55 [0.44–0.78] µg/mL FEU, *p* < 0.01), fibrinogen levels (7.08 [6.30–7.85] vs. 5.69 [4.90–6.06] g/L, *p* = 0.01), sTM concentrations (7206.20 [5514.25–10,352.00] vs. 4160.20 [3432.90–5104.10] pg/mL, *p* < 0.001), VWF:Ag levels (3.99 [3.38–4.83] vs. 3.20 [2.66–3.89] IU/mL, *p* = 0.03), and VWF:Ag/ADAMTS13 ratios (5.63 [4.83–8.98] vs. 3.39 [2.82–4.59], *p* < 0.01). ADAMTS13 activity was lower in patients with an unfavorable outcome (61.00 [51.50–83.50] vs. 85.50 [80.25–98.00] IU/dL, *p* < 0.01), whereas TFPI concentrations (60.00 [51.50–80.00] vs. 22.50 [17.00–37.25] ng/mL, *p* < 0.001), EV-TF activity (32.52 [5.00–74.71] vs. 5.00 [5.00–5.00] fM, *p* = 0.01), and EV-PGC (167.46 [70.67–425.89] vs. 36.66 [31.20–74.35] fM, *p* < 0.01) were higher. At day 7, an unfavorable outcome remained associated with higher fibrinogen levels (6.81 [5.70–8.59] vs. 4.90 [4.54–5.28] g/L, *p* = 0.01), VWF:Act (4.60 [3.90–5.16] vs. 3.17 [2.24–3.79] IU/mL, *p* < 0.01), VWF:Ag/ADAMTS13 ratios (7.92 [5.29–8.92] vs. 4.09 [3.65–4.88], *p* < 0.01), TFPI concentrations (62.00 [49.00–89.00] vs. 20.00 [16.00–23.00] ng/mL, *p* < 0.01), EV-TF activity (23.06 [7.65–78.00] vs. 5.00 [5.00–10.00] fM, *p* = 0.03), and EV-PGC (249.97 [100.82–499.87] vs. 76.31 [26.06–83.01] fM, *p* < 0.01). At day 7, sTM and VWF:Ag concentrations were numerically higher in patients with an unfavorable outcome (*p* = 0.052 and *p* = 0.051, respectively). These results are summarized in Tables S3 and S4, with selected biomarker profiles illustrated in Figure 4.

Additional exploratory analyses showed between-group differences in time-weighted biomarker exposure. According to initial disease severity, time-weighted exposures were higher in patients with severe COVID-19 for EV-PGC, EV-TF, soluble thrombomodulin, D-dimer, VWF:Act, VWF:Ag, the VWF:Ag/ADAMTS13 ratio, and TFPI, and lower for ADAMTS13 activity (all *p* ≤ 0.033). According to clinical outcome, patients with an unfavorable outcome had higher time-weighted exposures for EV-PGC, EV-TF, soluble thrombomodulin, fibrinogen, D-dimer, VWF:Act, VWF:Ag, the VWF:Ag/ADAMTS13 ratio, TFPI, and lower time-weighted ADAMTS13 and antithrombin activities (all *p* ≤ 0.037; Supplementary Table S7). In exploratory linear models with patient-specific fixed intercepts, nominal time-by-group interaction *p* values below 0.05 were observed according to initial disease severity for VWF:Act (*p* < 0.001), VWF:Ag (*p* = 0.027), ADAMTS13 activity (*p* = 0.043), and antithrombin activity (*p* = 0.004). According to clinical outcome, nominal interaction *p* values below 0.05 were observed for VWF:Act (*p* = 0.002), VWF:Ag (*p* = 0.047), and antithrombin activity (*p* < 0.001). The remaining biomarkers did not show nominal interaction *p* values below 0.05. These analyses were considered hypothesis-generating.

## 4. Discussion

This prospective study identified baseline differences in biomarkers spanning coagulation, endothelial function, endogenous antithrombotic regulation, and extracellular vesicle-mediated activity according to initial COVID-19 severity and clinical outcome. Several between-group differences were also detectable at days 3 and 7. Time-weighted mean levels and longitudinal analyses using models with patient-specific fixed intercepts provided additional exploratory information.

These findings are consistent with the involvement of multiple hemostatic and endothelial pathways in COVID-19-associated immunothrombosis [3,4,9,19]. The concurrent measurement of biomarkers spanning several biological pathways provides a broad multipathway description within the same patients. Biomarkers were analyzed separately, and no joint multivariable biomarker model was developed. Accordingly, these analyses do not determine whether individual markers provide independent or incremental information beyond routine clinical assessment, or whether combining them improves clinical discrimination.

### 4.1. Exploratory Discrimination by Admission Biomarkers

Exploratory ROC analyses illustrated the separation of baseline biomarker distributions between the predefined clinical groups, particularly for TFPI, EV-PGC, EV-TF, and D-dimer. These analyses were descriptive and did not assess the incremental clinical value of these biomarkers beyond the existing severity classification. The confidence intervals indicate uncertainty around the AUC estimates, and numerical differences do not establish superiority of one biomarker over another. Interpretation of the outcome analyses requires particular caution: 12 of the 17 patients with an unfavorable outcome were already receiving invasive mechanical ventilation at inclusion, and 16 belonged to the initially severe group. These analyses therefore combine baseline clinical status with subsequent events and substantially overlap with the severity comparison. They should not be interpreted as demonstrating prediction of subsequent clinical deterioration. The small sample size, variation in biomarker availability, and absence of internal or external validation further limit interpretation. Differences in heparin exposure may also have contributed to the apparent discrimination of TFPI. These exploratory associations warrant further investigation but do not establish independent prognostic value or clinical utility.

Endothelial perturbation also appeared to be a prominent component of this biological profile. Autopsy studies demonstrated diffuse pulmonary endothelialitis, widespread microvascular thrombosis, and extensive vascular remodeling, indicating that endothelial injury is a hallmark of severe SARS-CoV-2 infection rather than merely a secondary consequence of respiratory failure [3]. Mechanistic models of COVID-19-associated pulmonary intravascular coagulopathy have further emphasized that endothelial activation, leukocyte recruitment, platelet activation, and local thrombin generation act together within the pulmonary microcirculation [4]. Afzali et al. subsequently provided a broader integrated framework linking complement activation, endothelial injury, loss of thromboresistance, platelet activation, and coagulation activation, supporting the concept of a self-amplifying thrombo-inflammatory network rather than independent biological abnormalities [19].

Our observations were also consistent with an alteration of the VWF–ADAMTS13 axis in severe COVID-19. Systemic endothelial activation promotes VWF release, while reduced ADAMTS13 activity may impair VWF cleavage and favor platelet adhesion and microvascular thrombosis. Because Weibel–Palade body abundance varies across the vascular tree and is limited in parts of the pulmonary capillary endothelium, increased circulating VWF should primarily be interpreted as a marker of systemic endothelial activation rather than as evidence of release specifically from the pulmonary microcirculation. Mancini et al. reported a marked imbalance of the VWF–ADAMTS13 axis associated with COVID-19 severity, while Marco et al. similarly described increased VWF:Ag, reduced ADAMTS13 activity, and higher VWF:Ag/ADAMTS13 ratios in more severe patients [7,8]. The clinical relevance of this endothelial phenotype is further supported by meta-analytic evidence associating endothelial biomarkers, including the VWF–ADAMTS13 axis, with poor outcomes in COVID-19 [9].

Among the biomarkers assessed, the marked separation in TFPI concentrations between groups was noteworthy. Its marked increase contrasted with the lower antithrombin and protein C activities observed in the most severely affected patients, suggesting that endogenous anticoagulant pathways were not uniformly altered and warrant specific consideration.

### 4.2. Impairment of Endogenous Anticoagulant Pathways Contrasts with the Distinct TFPI Profile

Several biomarkers classified within endogenous anticoagulant pathways showed a pattern consistent with impaired antithrombotic regulation in the most severely affected patients. At baseline, ADAMTS13 and protein C activities were lower in patients with severe disease, while ADAMTS13, antithrombin, and protein C activities were lower in patients with an unfavorable outcome. Although ADAMTS13 is not a classical plasma anticoagulant such as antithrombin or protein C, its cleavage of VWF constitutes an endogenous antithrombotic regulatory mechanism. Free protein S antigen levels did not differ significantly between groups, but this antigenic measurement does not establish whether protein S functional activity was preserved. The protein C and antithrombin findings are consistent with those of Stanne et al., who reported that low protein C levels at admission were associated with disease worsening and mortality in hospitalized patients with COVID-19, and Boknäs et al., who found that low antithrombin activity was associated with excess mortality in COVID-19 [20,21].

Reduced antithrombin activity may weaken the inhibition of thrombin and factor Xa, whereas impairment of the protein C pathway may reduce the inactivation of factors Va and VIIIa. This imbalance may be reinforced by endothelial injury and thrombomodulin loss, which limit protein C activation. Consistently, Francischetti et al. demonstrated increased pulmonary tissue factor expression together with reduced thrombomodulin expression in fatal COVID-19, supporting a shift from an anticoagulant toward a procoagulant endothelial phenotype [22]. These abnormalities may result from overlapping mechanisms, including consumption during sustained coagulation activation, inflammatory proteolysis, altered synthesis, and vascular leakage, and may contribute to the persistence of the prothrombotic phenotype.

Free TFPI antigen concentrations were markedly higher in patients with severe COVID-19 and in those with an unfavorable outcome. Ueland et al. also reported higher circulating TFPI concentrations in patients with severe COVID-19 during hospitalization [23]. However, they measured total TFPI, whereas our study measured free TFPI antigen. Although both studies associate higher circulating TFPI concentrations with severe disease, this difference in the fractions measured limits direct comparison and prevents interpreting our findings as a direct replication.

In our cohort, interpretation of this finding is strongly limited by differential heparin exposure. TFPI inhibits the initiation of coagulation through factor Xa and the tissue factor–factor VIIa complex, and a substantial pool of endothelial-associated TFPI can be mobilized into the circulation by heparin [24]. Experimental studies have further shown that both unfractionated and low-molecular-weight heparin can promote the release and redistribution of TFPI by endothelial cells [25]. This mechanism is particularly relevant because actual anticoagulant exposure differed substantially between severity groups. Therapeutic anticoagulation was recorded in 15 patients with severe COVID-19 compared with six patients with moderate disease, and continuous intravenous unfractionated heparin was administered to 13 patients in the severe group but none in the moderate group. Chronic anticoagulation was also more frequent in the severe group, and blood sampling was not standardized according to the timing of anticoagulant administration. Differences in heparin type, dose, and timing may therefore have contributed substantially to both the observed TFPI concentrations and their high discriminative performance. In an exploratory sensitivity analysis restricted to patients receiving the same intensified prophylactic enoxaparin regimen, TFPI concentrations remained numerically higher in severe disease. However, the very small sample size and unavailable information on treatment duration and timing relative to blood sampling prevent drawing conclusions about an association independent of heparin exposure. Consequently, the respective contributions of disease-related endothelial perturbation and heparin-induced TFPI release cannot be distinguished in this cohort.

Elevated circulating TFPI may additionally reflect disease-related endothelial perturbation and mobilization of endothelial-associated TFPI. A compensatory anticoagulant response is also biologically plausible, but free TFPI antigen concentrations do not directly assess functional TFPI activity or effective local thromboresistance. TFPI should therefore be interpreted as a composite marker potentially reflecting endothelial perturbation, endogenous anticoagulant regulation, and heparin exposure rather than as a direct measure of effective anticoagulant capacity.

### 4.3. Extracellular Vesicle-Associated Procoagulant and Plasmin-Generating Activities

Beyond endothelial and endogenous anticoagulant abnormalities, our study highlights marked extracellular vesicle-associated hemostatic activation in severe COVID-19. Both EV-TF and EV-PGC were substantially higher at admission in patients with severe disease and in those with an unfavorable outcome. In the latter group, this pattern was accompanied by markedly increased D-dimer concentrations, suggesting simultaneous activation of coagulation, fibrin turnover, and extracellular vesicle-associated plasmin generation. In addition, both EV-TF and EV-PGC showed substantial between-group differences at baseline, which remained detectable in several comparisons performed at days 3 and 7. Together, these observations are compatible with a broad thrombo-inflammatory phenotype but do not establish mechanistic interactions between the measured pathways.

Extracellular vesicles released by activated platelets, leukocytes, and endothelial cells may expose negatively charged phospholipids, particularly phosphatidylserine, thereby providing catalytic surfaces for the assembly of coagulation enzyme complexes [6,26]. However, their procoagulant mechanisms differ according to cellular origin. Platelet-derived EVs are mainly procoagulant through phosphatidylserine exposure and are not generally considered a major endogenous source of TF. In contrast, circulating TF-bearing EVs are thought to originate predominantly from activated monocytes and, in inflammatory conditions, from endothelial cells. Platelet–leukocyte interactions may further contribute to the formation of EV populations displaying both platelet and leukocyte markers together with TF expression [14,16]. Thus, TF expression should not be considered a general property of all circulating EVs.

Our findings are consistent with previous reports of increased EV-TF activity in severe COVID-19. Guervilly et al. found that EV-TF activity was associated with disease severity and thrombotic risk [12], while Rosell et al. reported associations with severity and mortality [13]. The fibrinolytic findings require a distinction between comparators. In the study by Guervilly et al., higher EV-associated fibrinolytic activity without a parallel increase in PAI-1 was observed in severe COVID-19 compared with septic shock, whereas EV-associated fibrinolytic activity did not differ significantly between the moderate and severe COVID-19 groups [12]. Our finding of higher EV-PGC in severe than in moderate COVID-19 therefore extends, rather than directly replicates, those observations.

Higher EV-PGC indicates a greater capacity of circulating EVs to support plasmin generation under the assay conditions. It does not directly measure fibrin clearance in vivo or establish that fibrinolysis is sufficient relative to coagulation activation. Effective clot resolution also depends on factors not comprehensively assessed here, including plasminogen availability, plasminogen activators and their inhibitors, fibrin structure, and the anatomical distribution of fibrin deposition. Similarly, the absence of a between-group difference in PAI-1 concentrations neither establishes normal fibrinolytic regulation nor excludes impaired fibrinolysis in either group. The concurrent elevation of EV-TF and EV-PGC therefore documents increased procoagulant and plasmin-generating capacities associated with circulating EVs, while their net effect on fibrin formation and clearance remains uncertain.

Serial measurements showed that EV-TF remained higher in patients with initially severe disease at day 3, whereas the between-group difference in EV-PGC was no longer statistically significant. At day 7, both markers were numerically higher in this group, without statistically significant differences. Neither marker showed a significant time-by-group interaction. These findings therefore do not establish distinct temporal responses of EV-mediated procoagulant and plasmin-generating activity. Time-weighted mean levels of both markers were higher according to initial severity and unfavorable outcome, supporting differences over the available observation periods. However, variation in follow-up duration and in the patients contributing samples at each time point limits conclusions about within-patient trajectories or persistence throughout the first week.

### 4.4. Biological Interpretation of the Biomarker Panel

The panel was selected to cover several biological processes implicated in COVID-19-associated immunothrombosis. D-dimer reflects fibrin turnover; VWF-related markers, sTM, and ADAMTS13 inform different aspects of endothelial perturbation and VWF regulation; and antithrombin, protein C, protein S, and TFPI characterize components of endogenous antithrombotic regulation. EV-TF and EV-PGC provide functional measurements of EV-associated tissue factor activity and plasmin-generating capacity. Assessing these markers together documents the coexistence of differences across these processes, without demonstrating their mechanistic interdependence or statistical complementarity.

Repeated measurements extended this description beyond admission. Previous studies have also investigated serial hemostatic measurements in COVID-19. Juneja et al. reported associations between mortality and longitudinal profiles of sTM, PAI-1, plasminogen, and clot lysis time [27], while Campello et al. described changes in circulating EV profiles during and after hospitalization [28]. Our study adds concurrent assessment of EV-associated functional activities and a broader panel of hemostatic and endothelial biomarkers in patients with differing initial respiratory severity.

The inflammatory component of this phenotype also warrants consideration. In our cohort, patients with severe COVID-19 had higher baseline CRP concentrations and leukocyte counts than those with moderate disease (Table 1), consistent with a more pronounced systemic inflammatory response. Immunothrombosis involves reciprocal interactions between innate immunity, platelets, coagulation, and the vascular endothelium, and these processes have been implicated in COVID-19-associated microvascular and macrovascular thrombosis [29,30]. Beyond thrombotic complications, inflammatory indices have also been associated with clinical outcomes in COVID-19. Di Giorgio et al. reported associations between CRP and delirium, as well as between CRP or the neutrophil-to-lymphocyte ratio and ICU admission or death [31]. However, CRP and total leukocyte counts are nonspecific inflammatory markers, and our study did not directly examine relationships between inflammatory indices and hemostatic biomarkers. Therefore, although inflammatory and hemostatic abnormalities were concurrently observed in patients with severe disease, our findings do not establish mechanistic interactions between these pathways or their causal contribution to thrombotic complications.

Whether combining these measurements improves risk stratification or identifies reproducible biological phenotypes remains to be established. Larger studies should assess relationships between markers, their incremental information beyond clinical severity and routinely available measurements, and the reproducibility of any proposed biomarker combinations.

### 4.5. Strengths, Limitations and Perspectives

This study has several strengths. First, its prospective design allowed standardized assessment of a broad panel of biomarkers at baseline. Second, the simultaneous evaluation of coagulation activation, endothelial dysfunction, endogenous anticoagulant pathways, fibrinolytic regulation, and extracellular vesicle-mediated activity provided a broad multipathway biological description of COVID-19-associated immunothrombosis. Finally, predefined sampling at days 3 and 7 provided complementary secondary information on the early persistence of between-group differences.

Several limitations should also be acknowledged. This was a single-center exploratory study with a limited sample size, and no formal sample size calculation was performed. Moreover, the large number of unadjusted comparisons increases the risk of chance findings, and nominal *p* values should not be interpreted as confirmatory evidence or as demonstrating independent biomarker associations. The predefined inclusion of equal numbers of patients with moderate and severe disease may have improved between-group comparisons but limits generalizability to an unselected hospitalized COVID-19 population. In addition, the study was conducted during the early pre-vaccination phase of the pandemic, between April 2020 and January 2021, when circulating variants and therapeutic strategies differed from those of contemporary COVID-19 populations. Follow-up measurements were available only while patients remained hospitalized. Discharge and death could therefore alter the composition of the groups assessed at successive time points, and changes in group medians should not be interpreted as within-patient trajectories. Dividing concentration–time areas by the observed duration accounts for differences in interval length but does not make observation windows equivalent or remove bias from informative missingness. Similarly, longitudinal models with patient-specific fixed intercepts used all available measurements but did not eliminate the potential influence of outcome-related missing data.

Although several biomarkers reflected a prothrombotic biological phenotype, this study was not designed or powered to assess their association with clinical thrombotic events. Five thromboembolic events were documented in four patients at admission. However, imaging investigations were clinically indicated rather than systematic, and imaging data collected for this study were limited to admission. Therefore, these observations cannot be used to estimate the true incidence of thrombosis during hospitalization. The small number of events also precluded meaningful biomarker–thrombosis association analyses. In addition, the observational design precludes any causal inference regarding the contribution of the investigated biomarkers to disease progression. Finally, several assays, particularly extracellular vesicle-related measurements, remain technically specialized and are not yet widely available for routine clinical use.

Despite these limitations, our findings illustrate the descriptive value of simultaneously assessing biomarkers reflecting multiple pathways involved in COVID-19-associated immunothrombosis. Future studies should validate these baseline and serial cross-sectional findings in larger multicenter cohorts, assess their relevance in contemporary COVID-19 populations, and determine whether multipathway biomarker profiles could help identify biologically distinct thrombo-inflammatory phenotypes.

## 5. Conclusions

Severe COVID-19 and unfavorable outcomes were associated with baseline differences in biomarkers of coagulation, endothelial perturbation, endogenous antithrombotic regulation, and extracellular vesicle-mediated activity. Several between-group differences remained detectable at days 3 and 7 among patients with available measurements. Interpretation of TFPI concentrations is limited by differential heparin exposure. These exploratory findings describe a broad hemostatic profile associated with disease severity, while the added value of combining biomarkers requires evaluation in larger cohorts. Neither prognostic value for subsequent clinical deterioration nor causal relationships can be established from these observational data.

## Figures and Tables

**Table 1:** Baseline characteristics and patient outcomes.

	All Patients *n* = 42	Moderate COVID-19 *n* = 21	Severe COVID-19 *n* = 21	*p* Value
Age, years	58.50 [49.50–66.75]	58.00 [46.00–65.00]	59.00 [51.00–72.00]	0.70
Female, *n* (%)	18 (42.9)	10 (47.6)	8 (38.1)	0.53
BMI (kg/m^2^)	30.79 [26.56–33.80]	30.61 [28.13–32.49]	30.85 [26.06–34.40]	0.76
**Comorbidities, *n* (%)**				
Hypertension	17 (40.5)	7 (33.3)	10 (47.6)	0.35
Diabetes	11 (26.2)	4 (19.0)	7 (33.3)	0.29
Ischemic cardiomyopathy	5 (11.9)	2 (9.5)	3 (14.3)	1.00
Active smoking	0 (0)	0 (0)	0 (0)	—
COPD	8 (19.0)	5 (23.8)	3 (14.3)	0.70
VTE history	1 (2.4)	0 (0)	1 (4.8)	1.00
**Usual treatment, *n* (%)**				
Anticoagulation	5 (11.9)	0 (0)	5 (23.8)	<0.05
Antiplatelet treatment	10 (23.8)	4 (19.0)	6 (28.6)	0.47
**Clinical presentation at inclusion**				
Respiratory rate, cycles/min	24.00 [20.25–26.75]	24.00 [20.00–25.00]	24.00 [21.00–28.00]	0.53
SpO_2_, %	96.00 [94.25–97.00]	95.00 [94.00–97.00]	96.00 [95.00–98.00]	0.17
**Respiratory support at inclusion, *n* (%)**				<0.001
Room air	7 (16.7)	7 (33.3)	0 (0)	
Conventional oxygen supply	15 (35.7)	14 (66.7)	1 (4.8)	
High-flow nasal oxygen	5 (11.9)	0 (0)	5 (23.8)	
NIV + high-flow nasal oxygen	3 (7.1)	0 (0)	3 (14.3)	
Invasive ventilation	12 (28.6)	0 (0)	12 (57.1)	
SOFA score at inclusion	3 [1–4]	1 [1–2]	4 [3–7.25]	<0.001
**Arterial blood gas**				
pH	7.45 [7.40–7.47]	7.46 [7.45–7.47]	7.44 [7.36–7.47]	0.11
PaO_2_, mmHg	78.00 [69.75–94.25]	77.00 [68.50–90.00]	79.00 [71.00–97.00]	0.64
PaCO_2_, mmHg	35.00 [33.00–41.00]	34.00 [32.50–36.50]	37.00 [35.00–45.00]	0.02
Bicarbonates, mmol/L	24.00 [21.95–26.28]	24.00 [22.40–25.80]	24.00 [19.90–27.70]	0.99
Lactates, mmol/L	1.35 [1.00–1.73]	1.40 [1.00–1.55]	1.30 [1.00–1.90]	0.60
PaO_2/_FiO_2_, mmHg	266 [181–349]	329 [277–403]	180 [131–240]	<0.001
**SARS-CoV-2 RT-PCR Ct values**	27.00 [22.95–30.50] *n* = 31	29.00 [25.00–30.00] *n* = 17	24.50 [20.35–30.82] *n* = 14	0.16
**Chest pulmonary angiography** **performed**	33 (78.6)	21 (100)	12 (57.1)	<0.01
Chest CT findings, *n* = 33				
Ground-glass lesions < 50%	22 (66.7)	19 (90.5)	3 (25.0)	<0.001
Ground-glass lesions > 50%	11 (33.3)	2 (9.5)	9 (75.0)	<0.001
Consolidations	23 (69.7)	14 (66.7)	9 (75.0)	0.71
Crazy-paving patterns	9 (27.3)	5 (23.8)	4 (33.3)	0.69
Pleural effusion	2 (6.1)	2 (9.5)	0 (0)	0.52
**Biology at inclusion**				
Leukocytes count, G/L	7.05 [5.60–11.00]	6.10 [5.30–7.10]	8.30 [7.00–15.00]	0.01
Platelet count, G/L	251 [190–332]	245.5 [208–294]	270 [172–344]	0.80
Prothrombin activity, %	94.00 [85.00–101.00]	96.50 [93.00–106.50]	85.00 [70.00–99.00]	<0.01
APTT, ratio	1.20 [1.10–1.50]	1.10 [1.00–1.20]	1.40 [1.20–1.80]	<0.001
CRP, mg/L	143.30 [62.90–195.90]	78.30 [26.55–161.55]	179.95 [123.15–270.15]	<0.01
**Outcomes**				
Admission in ICU, *n* (%)	25 (59.5)	4 (19.0)	21 (100)	<0.001
Invasive ventilation, *n* (%)	16 (38.1)	0 (0)	16 (76.2)	<0.001
Death by day 28, *n* (%)	3 (7.1)	1 (4.8)	2 (9.5)	1.00
ICU length of stay, days	3.5 [0–15]	0 [0–0]	15 [9–28]	<0.001
Hospital length of stay, days	14.5 [8–26.75]	8 [6–14]	28 [15–32]	<0.001

Data are expressed as median [Q1–Q3] or number (%). Available sample sizes (moderate/severe) were 20/21 for BMI, platelet count and prothrombin activity; 19/21 for pH, PaO_2_, PaCO_2_, bicarbonate, lactate and APTT ratio; 19/19 for PaO_2_/FiO_2_; 17/20 for SOFA score; 19/18 for CRP; and 17/14 for SARS-CoV-2 RT-PCR Ct values. Other variables had 21/21 available observations, except chest CT findings, which were available in 21/12 patients. Definition of abbreviations: APTT, activated partial thromboplastin time; BMI, body mass index; COPD, chronic obstructive pulmonary disease; CRP, C-reactive protein; CT, computed tomography; Ct, cycle threshold; FiO_2_, fraction of inspired oxygen; ICU, intensive care unit; NIV, non-invasive ventilation; PaCO_2_, arterial partial pressure of carbon dioxide; PaO_2_, arterial partial pressure of oxygen; SOFA, Sequential Organ Failure Assessment; SpO_2_, peripheral oxygen saturation; VTE, venous thromboembolism.

**Table 2:** Biomarkers at admission according to initial disease severity.

Biomarker	Moderate COVID-19 (*n* = 21)	Severe COVID-19 (*n* = 21)	*p* Value
**Conventional coagulation activation**			
D-dimer, µg/mL FEU	0.68 [0.46–0.94]	1.74 [0.87–3.67]	<0.01
Fibrinogen, g/L	6.58 [5.77–7.31]	7.10 [5.99–7.84]	0.30
**Endothelial dysfunction**			
CEC, *n*/mL	3.00 [0.50–15.50]	5.00 [1.00–16.00]	0.71
sTM, pg/mL	3771.65 [3198.88–4459.42]	4826.60 [4031.45–7335.93]	<0.01
VWF:Act, IU/mL	2.70 [2.48–3.00]	3.20 [2.31–3.83]	0.32
VWF:Ag, IU/mL	3.18 [2.71–3.50]	4.05 [3.09–4.20]	<0.01
VWF:Ag/ADAMTS13 ratio	3.87 [2.87–4.72]	5.19 [3.77–7.96]	<0.01
Factor VIII, IU/mL	2.04 [1.62–2.29]	2.69 [1.97–3.05]	0.03
**Endogenous anticoagulant pathways**			
ADAMTS13 activity, IU/dL	85.00 [78.50–98.00]	69.00 [56.00–84.00]	<0.01
Antithrombin activity, %	102.00 [94.50–114.00]	96.00 [80.00–106.00]	0.06
Protein C activity, %	96.00 [84.50–115.00]	80.00 [75.00–107.00]	0.02
Free protein S antigen, %	79.00 [71.50–100.00]	79.00 [65.00–101.00]	0.66
Free TFPI antigen, ng/mL	16.00 [14.00–29.00]	55.00 [38.00–109.00]	<0.001
**Fibrinolysis**			
PAI-1, ng/mL	87.00 [73.00–100.50]	89.00 [61.00–141.00]	0.80
**Extracellular vesicle-mediated activity**			
EV-TF, fM	5.00 [5.00–5.70]	28.86 [13.61–48.72]	<0.001
EV-PGC, fM	24.50 [16.14–35.74]	93.36 [41.60–303.10]	<0.001

Data are expressed as median [Q1–Q3]. For differences between the 2 groups, the Mann–Whitney test was used. Column headers indicate the total number of patients in each clinical group; biomarker analyses were performed using available values, which varied by biomarker. For all biomarkers, the numbers of available measurements were 19/21 (moderate/severe), except for sTM (18/20), free TFPI (17/21), EV-TF (19/20), and EV-PGC (18/21). Definition of abbreviations: ADAMTS13, a disintegrin and metalloproteinase with thrombospondin type 1 motif, member 13; CEC, circulating endothelial cells; EV-PGC, extracellular vesicle-associated plasmin generation capacity; EV-TF, extracellular vesicle-associated tissue factor activity; PAI-1, plasminogen activator inhibitor-1; sTM, soluble thrombomodulin; TFPI, tissue factor pathway inhibitor; VWF:Act, von Willebrand factor activity; VWF:Ag, von Willebrand factor antigen.

**Table 3:** Biomarkers at admission according to clinical outcomes.

Biomarker	Favorable Outcome (*n* = 25)	Unfavorable Outcome (*n* = 17)	*p* Value
**Conventional coagulation activation**			
D-dimer, µg/mL FEU	0.60 [0.46–0.94]	1.98 [1.38–3.95]	<0.001
Fibrinogen, g/L	6.24 [5.51–7.21]	7.30 [6.21–8.80]	0.06
**Endothelial dysfunction**			
CEC, *n*/mL	3.00 [0.50–15.50]	5.00 [1.00–16.00]	0.68
sTM, pg/mL	3927.80 [3312.95–4644.65]	5665.35 [4119.95–9167.40]	<0.01
VWF:Act, IU/mL	2.68 [2.37–3.13]	3.20 [2.62–3.83]	0.18
VWF:Ag, IU/mL	3.09 [2.71–3.66]	4.05 [3.32–4.20]	0.01
VWF:Ag/ADAMTS13 ratio	3.72 [3.02–4.72]	5.65 [4.29–9.05]	<0.01
Factor VIII, IU/mL	2.12 [1.62–2.62]	2.57 [1.97–3.05]	0.08
**Endogenous anticoagulant pathways**			
ADAMTS13 activity, IU/dL	83.00 [75.50–98.00]	66.00 [47.00–88.00]	<0.01
Antithrombin activity, %	104.00 [96.50–115.50]	82.00 [73.00–99.00]	<0.01
Protein C activity, %	106.00 [84.50–115.00]	76.00 [66.00–96.00]	<0.01
Free protein S antigen, %	79.00 [68.50–100.00]	79.00 [65.00–101.00]	0.97
Free TFPI antigen, ng/mL	25.00 [14.00–32.00]	57.00 [40.00–112.00]	<0.001
**Fibrinolysis**			
PAI-1, ng/mL	89.00 [73.00–109.00]	86.00 [61.00–141.00]	0.99
**Extracellular vesicle-mediated activity**			
EV-TF, fM	5.00 [5.00–5.96]	30.27 [14.23–59.08]	<0.001
EV-PGC, fM	24.57 [16.59–42.06]	102.16 [66.62–376.05]	<0.001

Data are expressed as median [Q1–Q3]. For differences between the 2 groups, the Mann–Whitney test was used. Column headers indicate the total number of patients in each clinical group; biomarker analyses were performed using available values, which varied by biomarker. For all biomarkers, the numbers of available measurements were 23/17 (favorable/unfavorable), except for sTM (22/16), free TFPI (21/17), EV-TF (22/17), and EV-PGC (23/16). Definition of abbreviations: ADAMTS13, a disintegrin and metalloproteinase with thrombospondin type 1 motif, member 13; CEC, circulating endothelial cells; EV-PGC, extracellular vesicle-associated plasmin generation capacity; EV-TF, extracellular vesicle-associated tissue factor activity; PAI-1, plasminogen activator inhibitor-1; sTM, soluble thrombomodulin; TFPI, tissue factor pathway inhibitor; VWF:Act, von Willebrand factor activity; VWF:Ag, von Willebrand factor antigen.

## Data Availability

All data are available upon reasonable request.

## Supplementary Materials

The following supporting information can be downloaded at: https://www.mdpi.com/article/10.3390/jcm15218444/s1, Table S1: Biomarkers at day 3 according to initial disease severity; Table S2: Biomarkers at day 7 according to initial disease severity; Table S3: Biomarkers at day 3 according to clinical outcome; Table S4: Biomarkers at day 7 according to clinical outcome; Table S5: AUC values on days 3 and 7 according to initial disease severity; Table S6: AUC values on days 3 and 7 according to clinical outcome; Table S7: Biomarkers with nominal *p* values below 0.05 in comparisons of time-weighted exposure during the available follow-up; Table S8: Baseline biomarkers according to subsequent clinical outcome in patients not receiving invasive mechanical ventilation at the first blood sample; Table S9. Documented anticoagulant exposure at study visits according to initial disease severity.

## Abbreviations

The following abbreviations are used in this manuscript:

ADAMTS13	A Disintegrin And Metalloproteinase with ThromboSpondin type 1 motif, member 13
APTT	Activated Partial Thromboplastin Time
AUC	Area Under the Receiver Operating Characteristic Curve
BMI	Body Mass Index
CEC	Circulating Endothelial Cells
COPD	Chronic Obstructive Pulmonary Disease
COVID-19	Coronavirus Disease 2019
CRP	C-Reactive Protein
CT	Computed Tomography
Ct	Cycle threshold
ELISA	Enzyme-Linked ImmunoSorbent Assay
EV	Extracellular Vesicle
EV-PGC	Extracellular Vesicle-associated Plasmin Generation Capacity
EV-TF	Extracellular Vesicle-associated Tissue Factor activity
FiO_2_	Fraction of inspired Oxygen
FXa	Activated Factor X
ICU	Intensive Care Unit
LMWH	Low-Molecular-Weight Heparin
NIV	Non-Invasive Ventilation
PaCO_2_	Arterial Partial pressure of Carbon Dioxide
PaO_2_	Arterial Partial pressure of Oxygen
PAI-1	Plasminogen Activator Inhibitor-1
ROC	Receiver Operating Characteristic
RT-PCR	Reverse Transcription Polymerase Chain Reaction
SARS-CoV-2	Severe Acute Respiratory Syndrome Coronavirus 2
SOFA	Sequential Organ Failure Assessment
SpO_2_	Peripheral Oxygen Saturation
sTM	Soluble ThromboModulin
TF	Tissue Factor
TFPI	Tissue Factor Pathway Inhibitor
VTE	Venous ThromboEmbolism
VWF	Von Willebrand Factor
VWF:Act	Von Willebrand Factor Activity
VWF:Ag	Von Willebrand Factor Antigen

## Appendix A

### Methods

Eight 5 mL venous blood samples were collected at each time point: five tubes containing 0.129 M trisodium citrate, two EDTA-containing tubes, and one tube without additive.

The following biomarkers were assayed at the hematology laboratory of La Timone Hospital, in Marseille, France: prothrombin activity, APTT, VWF:Ag, VWF:Act, factor VIII, ADAMTS13, fibrinogen, antithrombin, protein C, protein S, TFPI, D-dimer and PAI-1.

Prothrombin activity measured with Neoptimal reagent (Stago, Asnières-sur-Seine, France) on STAR device.

VWF:Ag: measured with Liatest vWF:Ag reagent (Stago, Asnières-sur-Seine, France) on STAR device.

VWF:Act: measured with HemosIL reagent (Werfen, Barcelona, Spain) on Acustar device.

Factor VIII: measured with Immunodef VIII and CKprest reagents (Stago, Asnières-sur-Seine, France) on STAR device.

ADAMTS13: measured by ELISA method Technozyme ADAMTS-13 Activity reagent (Technoclone, Vienna, Austria).

Fibrinogen: measured with Liquid-Fib reagent (Stago, Asnières-sur-Seine, France) on STAR device.

Antithrombin: measured with Stachrom Antithrombin reagent (Stago, Asnières-sur-Seine, France) on STAR device.

Protein C: measured with Biophen Protein C reagent (Hyphen BioMed, Neuville-sur-Oise, France) on STAR device.

Free protein S antigen: measured using STA-Liatest Free Protein S reagent (Stago, Asnières-sur-Seine, France) on a STA-R analyzer.

TFPI: measured by ELISA method using Free TFPI antigen reagent (Stago, Asnières-sur-Seine, France).

D-dimer: measured with Liatest D-Di Plus reagent (Stago, Asnières-sur-Seine, France) on STAR device.

PAI-1: measured by ELISA method using Asserachrom PAI-1 (Stago, Asnières-sur-Seine, France).

The following biomarkers were assayed at the hematology laboratory of La Conception Hospital in Marseille, France: CEC, EV-TF and sTM.

CEC were identified according to previously published approaches for circulating endothelial cell characterization [32].

Extracellular vesicle-associated tissue factor activity (EV-TF) was measured using a TF-dependent FXa generation assay described in [17].

sTM was measured by ELISA method with Quantikine Human thrombomodulin/BDCA-3 reagent (Bio-Techne, Minneapolis, MN, USA).
