# Supplementary material for: Multipathway Biomarker Profiling of Immunothrombosis in Moderate and Severe COVID-19
Source: J Clin Med. 2026 Oct 29;15(21):8444. doi: 10.3390/jcm15218444 (PMC13763991; doi:10.3390/jcm15218444)
Supplement: Supplementary file 1 [file jcm-15-08444-s001.zip › Table S2.pdf]

Table S2. Biomarkers at day 7 according to initial disease severity

Biomarker	Moderate COVID-19 (n=13)	Severe COVID-19 (n=20)	p value
**Conventional coagulation activation**			
D-dimer, µg/mL FEU	0.87 [0.35–1.94]	2.55 [1.66–3.50]	0.03
Fibrinogen, g/L	5.07 [4.51–5.47]	6.79 [5.52–8.54]	0.046
**Endothelial dysfunction**			
CEC, n/mL	3.00 [1.00–6.00]	6.00 [0.00–15.00]	0.50
sTM, pg/mL	5040.00 [4326.40–6463.30]	7258.10 [5570.20–10195.85]	0.10
VWF:Act, IU/mL	2.43 [2.10–3.27]	4.58 [3.98–5.15]	<0.01
VWF:Ag, IU/mL	2.84 [2.73–3.16]	4.71 [4.20–6.30]	<0.01
VWF:Ag/ADAMTS13 ratio	4.37 [3.63–5.01]	7.15 [4.82–8.63]	0.07
Factor VIII, IU/mL	2.79 [2.58–2.93]	3.09 [2.54–3.71]	0.16
**Endogenous anticoagulant pathways**			
ADAMTS13 activity, IU/dL	79.00 [67.00–87.00]	73.50 [61.75–91.50]	0.95
Antithrombin activity, %	102.00 [91.00–110.00]	96.00 [89.75–105.75]	0.65
Protein C activity, %	126.00 [101.50–156.00]	115.00 [97.00–128.00]	0.24
Free protein S antigen, %	91.00 [86.50–101.00]	98.00 [87.00–104.50]	0.67
Free TFPI antigen, ng/mL	16.00 [14.25–19.25]	61.00 [41.50–88.00]	<0.001
**Fibrinolysis**			
PAI-1, ng/mL	73.00 [67.00–136.50]	81.50 [62.50–107.50]	0.67
**Extracellular vesicle-mediated activity**			
EV-TF, fM	6.33 [5.00–12.94]	22.96 [5.00–74.85]	0.14
EV-PGC, fM	80.97 [65.78–303.52]	209.78 [91.82–467.06]	0.23

Data are expressed as median [Q1–Q3]. For differences between the 2 groups, the Mann–Whitney test was used. Column headers indicate the number of patients with a documented day 7 study visit, irrespective of biomarker availability. Biomarker analyses were performed using available values, and the exact number of observations may therefore vary according to the biomarker. Analyzed sample sizes were 7/18 (moderate/severe) for all biomarkers except CEC (7/17), sTM (8/18), factor VIII (6/16), free TFPI (6/18), EV-TF (8/18), and EV-PGC (8/18).

Definition of abbreviations: ADAMTS13, a disintegrin and metalloproteinase with thrombospondin type 1 motif, member 13; CEC, circulating endothelial cells; EV-PGC, extracellular vesicle-associated plasmin generation capacity; EV-TF, extracellular vesicle-associated tissue factor activity; PAI-1, plasminogen activator inhibitor-1; sTM, soluble thrombomodulin; TFPI, tissue factor pathway inhibitor; VWF:Act, von Willebrand factor activity; VWF:Ag, von Willebrand factor antigen.
