# Supplementary material for: Multipathway Biomarker Profiling of Immunothrombosis in Moderate and Severe COVID-19
Source: J Clin Med. 2026 Oct 29;15(21):8444. doi: 10.3390/jcm15218444 (PMC13763991; doi:10.3390/jcm15218444)
Supplement: Supplementary file 1 [file jcm-15-08444-s001.zip › Table S3.pdf]

Table S3. Biomarkers at day 3 according to clinical outcome

Biomarker	Favorable outcome (n=25)	Unfavorable outcome (n=17)	p value
**Conventional coagulation activation**			
D-dimer, µg/mL FEU	0.55 [0.44–0.78]	1.42 [0.88–3.52]	<0.01
Fibrinogen, g/L	5.69 [4.90–6.06]	7.08 [6.30–7.85]	0.01
**Endothelial dysfunction**			
CEC, n/mL	1.00 [0.00–4.00]	7.00 [0.00–12.50]	0.32
sTM, pg/mL	4160.20 [3432.90–5104.10]	7206.20 [5514.25–10352.00]	<0.001
VWF:Act, IU/mL	2.69 [2.22–3.66]	3.30 [3.04–4.33]	0.09
VWF:Ag, IU/mL	3.20 [2.66–3.89]	3.99 [3.38–4.83]	0.03
VWF:Ag/ADAMTS13 ratio	3.39 [2.82–4.59]	5.63 [4.83–8.98]	<0.01
Factor VIII, IU/mL	2.60 [2.12–2.87]	3.02 [2.28–3.92]	0.24
**Endogenous anticoagulant pathways**			
ADAMTS13 activity, IU/dL	85.50 [80.25–98.00]	61.00 [51.50–83.50]	<0.01
Antithrombin activity, %	101.00 [94.50–114.00]	99.00 [90.50–113.00]	0.61
Protein C activity, %	128.00 [110.00–134.75]	109.00 [96.50–136.50]	0.29
Free protein S antigen, %	97.50 [84.75–108.75]	97.00 [82.00–105.50]	0.76
Free TFPI antigen, ng/mL	22.50 [17.00–37.25]	60.00 [51.50–80.00]	<0.001
**Fibrinolysis**			
PAI-1, ng/mL	102.00 [73.25–130.75]	92.00 [63.50–148.50]	0.91
**Extracellular vesicle-mediated activity**			
EV-TF, fM	5.00 [5.00–5.00]	32.52 [5.00–74.71]	0.01
EV-PGC, fM	36.66 [31.20–74.35]	167.46 [70.67–425.89]	<0.01

Data are expressed as median [Q1–Q3]. For differences between the 2 groups, the Mann–Whitney test was used. Column headers indicate the number of patients who remained hospitalized at the corresponding time point. Biomarker analyses were performed using available values, and the exact number of observations may therefore vary according to the biomarker. Analyzed sample sizes were 18/15 (favorable/unfavorable) for all biomarkers except CEC (17/15), sTM (17/15), factor VIII (18/14), free TFPI (16/15), EV-TF (17/16), and EV-PGC (17/16).

Definition of abbreviations: ADAMTS13, a disintegrin and metalloproteinase with thrombospondin type 1 motif, member 13; CEC, circulating endothelial cells; EV-PGC, extracellular vesicle-associated plasmin generation capacity; EV-TF, extracellular vesicle-associated tissue factor activity; PAI-1, plasminogen activator inhibitor-1; sTM, soluble thrombomodulin; TFPI, tissue factor pathway inhibitor; VWF:Act, von Willebrand factor activity; VWF:Ag, von Willebrand factor antigen.
