# Supplementary material for: Multipathway Biomarker Profiling of Immunothrombosis in Moderate and Severe COVID-19
Source: J Clin Med. 2026 Oct 29;15(21):8444. doi: 10.3390/jcm15218444 (PMC13763991; doi:10.3390/jcm15218444)
Supplement: Supplementary file 1 [file jcm-15-08444-s001.zip › Table S4.pdf]

Table S4. Biomarkers at day 7 according to clinical outcome

Biomarker	Favorable outcome (n=16)	Unfavorable outcome (n=17)	p value
**Conventional coagulation activation**			
D-dimer, µg/mL FEU	0.79 [0.36–2.60]	2.42 [1.65–3.54]	0.07
Fibrinogen, g/L	4.90 [4.54–5.28]	6.81 [5.70–8.59]	0.01
**Endothelial dysfunction**			
CEC, n/mL	2.50 [0.00–5.00]	7.50 [2.25–17.25]	0.24
sTM, pg/mL	5550.40 [4226.80–6406.90]	8004.10 [5502.70–10526.00]	0.052
VWF:Act, IU/mL	3.17 [2.24–3.79]	4.60 [3.90–5.16]	<0.01
VWF:Ag, IU/mL	3.16 [2.82–4.00]	4.64 [4.20–6.30]	0.051
VWF:Ag/ADAMTS13 ratio	4.09 [3.65–4.88]	7.92 [5.29–8.92]	<0.01
Factor VIII, IU/mL	2.93 [2.60–2.94]	3.02 [2.48–3.81]	0.44
**Endogenous anticoagulant pathways**			
ADAMTS13 activity, IU/dL	79.50 [72.50–95.00]	68.00 [60.00–90.00]	0.20
Antithrombin activity, %	105.50 [99.25–109.50]	96.00 [85.00–105.00]	0.13
Protein C activity, %	124.00 [102.25–152.50]	114.00 [99.00–129.00]	0.26
Free protein S antigen, %	90.50 [86.00–100.75]	99.00 [87.00–105.00]	0.56
Free TFPI antigen, ng/mL	20.00 [16.00–23.00]	62.00 [49.00–89.00]	<0.01
**Fibrinolysis**			
PAI-1, ng/mL	70.50 [62.00–142.75]	83.00 [64.00–109.00]	0.98
**Extracellular vesicle-mediated activity**			
EV-TF, fM	5.00 [5.00–10.00]	23.06 [7.65–78.00]	0.03
EV-PGC, fM	76.31 [26.06–83.01]	249.97 [100.82–499.87]	<0.01

Data are expressed as median [Q1–Q3]. For differences between the 2 groups, the Mann–Whitney test was used. Column headers indicate the number of patients with a documented day 7 study visit, irrespective of biomarker availability. Biomarker analyses were performed using available values, and the exact number of observations may therefore vary according to the biomarker. Analyzed sample sizes were 8/17 (favorable/unfavorable) for all biomarkers except CEC (8/16), sTM (9/17), factor VIII (7/15), free TFPI (7/17), EV-TF (9/17), and EV-PGC (9/17).

Definition of abbreviations: ADAMTS13, a disintegrin and metalloproteinase with thrombospondin type 1 motif, member 13; CEC, circulating endothelial cells; EV-PGC, extracellular vesicle-associated plasmin generation capacity; EV-TF, extracellular vesicle-associated tissue factor activity; PAI-1, plasminogen activator

inhibitor-1; sTM, soluble thrombomodulin; TFPI, tissue factor pathway inhibitor; VWF:Act, von Willebrand factor activity; VWF:Ag, von Willebrand factor antigen.
