# Supplementary material for: Multipathway Biomarker Profiling of Immunothrombosis in Moderate and Severe COVID-19
Source: J Clin Med. 2026 Oct 29;15(21):8444. doi: 10.3390/jcm15218444 (PMC13763991; doi:10.3390/jcm15218444)
Supplement: Supplementary file 1 [file jcm-15-08444-s001.zip › Table S6.pdf]

Table S6. AUCs and 95% confidence intervals on days 3 and 7 according to clinical outcome

**Biomarker**	**Day 3,** n (favorable/unfavorable)	**Day 3 AUC [95% CI]**	**Day 7,** n (favorable/unfavorable)	**Day 7 AUC [95% CI]**
**Conventional coagulation activation**				
D-dimer, μg/mL FEU	33 (18/15)	0.802 [0.648–0.956]	25 (8/17)	0.735 [0.497–0.973]
Fibrinogen, g/L	33 (18/15)	0.759 [0.577–0.941]	25 (8/17)	0.820 [0.635–1.000]
**Endothelial dysfunction**				
CEC, n/mL	32 (17/15)	0.602 [0.399–0.805]	24 (8/16)	0.652 [0.418–0.887]
sTM, pg/mL	32 (17/15)	0.902 [0.797–1.000]	26 (9/17)	0.739 [0.539–0.938]
VWF:Act, IU/mL	33 (18/15)	0.678 [0.488–0.867]	25 (8/17)	0.824 [0.658–0.989]
VWF:Ag, IU/mL	33 (18/15)	0.722 [0.546–0.899]	25 (8/17)	0.750 [0.550–0.950]
VWF:Ag/ADAMTS13 ratio	33 (18/15)	0.830 [0.686–0.973]	25 (8/17)	0.853 [0.701–1.000]
Factor VIII, IU/mL	32 (18/14)	0.625 [0.417–0.833]	22 (7/15)	0.610 [0.368–0.851]
**Endogenous anticoagulant pathways**				
ADAMTS13 activity, IU/dL	33 (18/15)	0.213 [0.036–0.390]	25 (8/17)	0.335 [0.114–0.555]
Antithrombin activity, %	33 (18/15)	0.446 [0.234–0.658]	25 (8/17)	0.305 [0.084–0.526]
Protein C activity, %	33 (18/15)	0.391 [0.189–0.593]	25 (8/17)	0.353 [0.099–0.607]
Free protein S antigen, %	33 (18/15)	0.467 [0.261–0.673]	25 (8/17)	0.577 [0.313–0.842]
Free TFPI antigen, ng/mL	31 (16/15)	0.869 [0.730–1.000]	24 (7/17)	0.929 [0.816–1.000]
**Fibrinolysis**				
PAI-1, ng/mL	33 (18/15)	0.487 [0.276–0.698]	25 (8/17)	0.507 [0.212–0.802]
**Extracellular vesicle-mediated activity**				
EV-TF, fM	33 (17/16)	0.739 [0.564–0.914]	26 (9/17)	0.758 [0.562–0.954]
EV-PGC, fM	33 (17/16)	0.772 [0.594–0.950]	26 (9/17)	0.837 [0.643–1.000]

Data are expressed as areas under the receiver operating characteristic curve (AUCs) with 95% confidence intervals (95% CIs) estimated using DeLong's method. AUCs were calculated using available biomarker measurements at each time point. The exact numbers of patients included in each analysis, overall and by clinical group, are reported in the corresponding columns. For all analyses, severe disease or unfavorable outcome was defined as the positive class, with higher biomarker values consistently assigned to the positive direction. AUC values below 0.5 therefore indicate an association of lower biomarker values with the positive class.
Definition of abbreviations: ADAMTS13, a disintegrin and metalloproteinase with thrombospondin type 1 motif, member 13; CEC, circulating endothelial cells; EV-PGC, extracellular vesicle-associated plasmin generation capacity; EV-TF, extracellular vesicle-associated tissue factor activity; PAI-1, plasminogen activator inhibitor-1; AUC, area under the receiver operating characteristic curve; sTM, soluble thrombomodulin; TFPI, tissue factor pathway inhibitor; VWF:Act, von Willebrand factor activity; VWF:Ag, von Willebrand factor antigen.
