# Supplementary material for: Multipathway Biomarker Profiling of Immunothrombosis in Moderate and Severe COVID-19
Source: J Clin Med. 2026 Oct 29;15(21):8444. doi: 10.3390/jcm15218444 (PMC13763991; doi:10.3390/jcm15218444)
Supplement: Supplementary file 1 [file jcm-15-08444-s001.zip › Table S7.pdf]

Table S7. Biomarkers with nominal p values below 0.05 in comparisons of time-weighted exposure during the available follow-up

Panel A. Initial disease severity			
**Biomarker**	**Moderate COVID-19 (n=21)**	**Severe COVID-19 (n=21)**	**p value**
D-dimer, µg/mL FEU	0.81 [0.48–1.09]	1.79 [1.07–3.62]	0.009
sTM, pg/mL	3948.93 [3271.13–5084.62]	5794.50 [5138.74–9096.13]	0.002
VWF:Act, IU/mL	2.91 [2.26–3.30]	3.81 [2.93–4.65]	0.007
VWF:Ag, IU/mL	2.98 [2.84–3.63]	4.27 [3.57–5.01]	0.002
VWF:Ag/ADAMTS13 ratio	3.70 [3.02–4.58]	5.79 [4.59–8.48]	0.007
ADAMTS13 activity, IU/dL	83.75 [77.38–95.88]	71.33 [62.33–90.63]	0.033
Free TFPI antigen, ng/mL	20.08 [17.00–23.75]	60.50 [47.33–84.67]	<0.001
EV-TF, fM	5.62 [5.00–15.85]	31.28 [23.57–98.88]	<0.001
EV-PGC, fM	46.58 [27.88–74.61]	175.74 [63.61–298.87]	0.032
**Panel B. Clinical outcome**			
**Biomarker**	**Favorable outcome (n=25)**	**Unfavorable outcome (n=17)**	**p value**
D-dimer, µg/mL FEU	0.74 [0.46–1.09]	1.82 [1.44–4.36]	<0.001
Fibrinogen, g/L	5.78 [5.55–6.59]	6.94 [6.15–8.05]	0.014
sTM, pg/mL	3948.93 [3322.30–5112.38]	6960.72 [5253.95–9337.40]	<0.001
VWF:Act, IU/mL	2.93 [2.35–3.52]	3.82 [3.05–4.59]	0.013
VWF:Ag, IU/mL	2.98 [2.83–3.74]	4.30 [3.71–4.95]	0.004
VWF:Ag/ADAMTS13 ratio	3.67 [3.23–4.65]	6.41 [5.02–8.68]	<0.001
ADAMTS13 activity, IU/dL	81.50 [77.00–91.00]	66.83 [53.67–90.50]	0.008
Antithrombin activity, %	109.00 [97.17–113.50]	92.00 [88.67–103.50]	0.037
Free TFPI antigen, ng/mL	23.00 [17.75–34.50]	58.50 [48.50–85.17]	<0.001
EV-TF, fM	6.32 [5.00–19.23]	49.38 [26.12–109.02]	<0.001
EV-PGC, fM	44.68 [27.26–69.21]	236.56 [149.17–472.22]	<0.001

Data are expressed as median [Q1–Q3] of time-weighted mean biomarker levels. Time-weighted means were calculated by dividing the trapezoidal area under the biomarker–time curve by the interval between the first and last available measurements. Only patients with at least two available measurements for the corresponding biomarker were included. Column headers indicate the total number of patients in each clinical group; the number included in each analysis varied according to biomarker availability. In Panel A, analyzed sample sizes were 14/20 (moderate/severe) for all biomarkers except sTM (15/19), free TFPI (12/20), EV-TF (15/20), and EV-PGC (15/20). In Panel B, analyzed sample sizes were 17/17 (favorable/unfavorable) for all biomarkers except free TFPI (15/17), EV-TF (18/17), and EV-PGC (18/17). Comparisons were performed using the Mann–Whitney U

test. For each comparison, only biomarkers with a nominal p value < 0.05 are shown.
Definition of abbreviations: ADAMTS13, a disintegrin and metalloproteinase with thrombospondin type 1 motif, member 13; CEC, circulating endothelial cells; EV-PGC, extracellular vesicle-associated plasmin generation capacity; EV-TF, extracellular vesicle-associated tissue factor activity; PAI-1, plasminogen activator inhibitor-1; sTM, soluble thrombomodulin; TFPI, tissue factor pathway inhibitor; VWF:Act, von Willebrand factor activity; VWF:Ag, von Willebrand factor antigen.
