# Supplementary material for: Multipathway Biomarker Profiling of Immunothrombosis in Moderate and Severe COVID-19
Source: J Clin Med. 2026 Oct 29;15(21):8444. doi: 10.3390/jcm15218444 (PMC13763991; doi:10.3390/jcm15218444)
Supplement: Supplementary file 1 [file jcm-15-08444-s001.zip › Table S8.pdf]

Table S8. Baseline biomarkers according to subsequent clinical outcome in patients not receiving invasive mechanical ventilation at the first blood sample

Biomarker	No documented subsequent event (n=25)	Subsequent composite event (n=5)
**Conventional coagulation activation**		
D-dimer, µg/mL FEU	0.60 [0.46–0.94]	1.38 [0.87–1.45]
Fibrinogen, g/L	6.24 [5.51–7.21]	6.36 [6.21–6.82]
**Endothelial dysfunction**		
CEC, n/mL	3.00 [0.50–15.50]	5.00 [1.00–16.00]
sTM, pg/mL	3927.80 [3312.95–4644.65]	5223.70 [4355.23–6205.17]
VWF:Act, IU/mL	2.68 [2.37–3.13]	2.00 [1.84–2.95]
VWF:Ag, IU/mL	3.09 [2.71–3.66]	2.99 [2.58–3.18]
VWF:Ag/ADAMTS13 ratio	3.72 [3.02–4.72]	4.61 [3.33–4.67]
Factor VIII, IU/mL	2.12 [1.62–2.62]	1.97 [1.81–2.57]
**Endogenous anticoagulant pathways**		
ADAMTS13 activity, IU/dL	83.00 [75.50–98.00]	60.00 [56.00–90.00]
Antithrombin activity, %	104.00 [96.50–115.50]	86.00 [79.00–99.00]
Protein C activity, %	106.00 [84.50–115.00]	75.00 [68.00–96.00]
Free protein S antigen, %	79.00 [68.50–100.00]	101.00 [79.00–102.00]
Free TFPI antigen, ng/mL	25.00 [14.00–32.00]	40.00 [38.00–57.00]
**Fibrinolysis**		
PAI-1, ng/mL	89.00 [73.00–109.00]	86.00 [52.00–93.00]
**Extracellular vesicle-mediated activity**		
EV-TF, fM	5.00 [5.00–5.96]	14.16 [5.00–26.31]
EV-PGC, fM	24.57 [16.59–42.06]	43.09 [25.88–59.73]

Data are expressed as median [Q1–Q3]. Column headers indicate the total number of patients in each group. Analyzed sample sizes were 23/5 (no documented subsequent event/subsequent composite event) for all biomarkers except sTM (22/4), free TFPI (21/5), EV-TF (22/5), and EV-PGC (23/4). This post hoc exploratory analysis excluded the 12 patients already receiving invasive mechanical ventilation at the first blood sample. Five patients subsequently reached the composite endpoint: four required invasive mechanical ventilation, including one who subsequently died, and one died without invasive mechanical ventilation. Given the small number of events, the analysis was descriptive, without hypothesis tests or ROC analyses. Missing biomarker values were not imputed.

Definition of abbreviations: ADAMTS13, a disintegrin and metalloproteinase with thrombospondin type 1 motif, member 13; CEC, circulating endothelial cells; EV-PGC, extracellular vesicle-associated plasmin generation capacity; EV-TF, extracellular vesicle-associated tissue factor activity; PAI-1, plasminogen activator inhibitor-1; sTM, soluble thrombomodulin; TFPI, tissue factor pathway inhibitor; VWF:Act, von Willebrand factor activity; VWF:Ag, von Willebrand factor antigen.
