# Supplementary material for: Multipathway Biomarker Profiling of Immunothrombosis in Moderate and Severe COVID-19
Source: J Clin Med. 2026 Oct 29;15(21):8444. doi: 10.3390/jcm15218444 (PMC13763991; doi:10.3390/jcm15218444)
Supplement: Supplementary file 1 [file jcm-15-08444-s001.zip › Table S9.pdf]

Table S9. Documented anticoagulant exposure at study visits according to initial disease severity

Variable	Day 1 moderate (n=21)	Day 1 severe (n=21)	Day 3 moderate (n=21)	Day 3 severe (n=21)	Day 7 moderate (n=21)	Day 7 severe (n=21)
**Standard-dose prophylaxis**	8	1	6	2	2	2
enoxaparin	8	1	6	2	2	2
**Intensified prophylaxis**	7	3	11	3	8	2
enoxaparin	7	3	10	3	8	2
subcutaneous UFH	0	0	1	0	0	0
**Therapeutic anticoagulation**	6	15	4	16	2	16
enoxaparin	3	2	2	3	0	5
tinzaparin	2	0	2	0	2	0
UFH	1	13	0	13	0	11
**Anticoagulant regimen not documented**	0	2	0	0	9	1
**Venovenous ECMO**	0	2	0	3	0	2

Data are expressed as numbers of patients. Treatments are classified by anticoagulant agent and prescribed intensity at each study visit; the denominators are 21 patients per initial severity group. Unfractionated heparin (UFH) was administered by continuous intravenous infusion in the severe group at baseline. Among the 13 severe patients with therapeutic UFH recorded at day 3, one dose entry did not establish the route of administration. The category "Anticoagulant regimen not documented" includes both visits with no recorded anticoagulant regimen and visits that were not performed. This category does not establish absence of anticoagulation. Treatment duration before sampling and the interval from the most recent dose to blood collection were unavailable. Venovenous extracorporeal membrane oxygenation is reported separately and may overlap with anticoagulant treatment categories. Definition of abbreviations: ECMO, extracorporeal membrane oxygenation; UFH, unfractionated heparin.
